# Decoupling pitch and angle of attack with a variable-incidence wing extends quadrotor flight endurance

**DOI:** 10.1038/s41598-026-58543-6

**Published:** 2026-06-18

**Authors:** Chengchen Shentu, Sanku Niu, Tao Wang, Jiaming Hu, Jun Wang

**Affiliations:** 1 Zhejiang Key Laboratory of Intelligent Manufacturing for Aerodynamic Equipment, Quzhou University, No.78, North Jiuhua Road, Quzhou, 324000 China; 2 Quzhou Institute of Special Equipment Inspection and Testing, No. 385 Tingchuan East Road, Kecheng District, Quzhou, 324000 China; 3 College of Mechanical Engineering, Zhejiang University of Technology, No.18, Chaowang Road, Hangzhou, 310014 Zhejiang Province China

**Keywords:** Aerodynamic interference, Variable-incidence wing, Energy efficiency, Quadrotor UAV, CFD simulation, Flight validation., Engineering, Physics

## Abstract

The aerodynamic efficiency of conventional lifting-wing quadrotors is severely constrained by the rigid coupling between fuselage pitch attitude and wing angle of attack (AoA), which prevents the vehicle from maintaining optimal lift-to-drag performance across varying flight speeds. This study presents a novel variable-incidence lifting-wing quadrotor that effectively decouples fuselage attitude from wing aerodynamics. The proposed mechanism incorporates a 143 g lightweight lifting wing and a dual-actuator drive system, with a total structural mass penalty of only 209 g. High-fidelity Computational Fluid Dynamics (CFD) simulations, reveal that the variable-incidence strategy alleviates detrimental rotor-wing interference. Specifically, a fuselage pitch of − 20° combined with a wing AoA of 10°–12° reestablishes a strong suction peak on the wing’s upper surface, maximizing passive lift. Systematic flight experiments validate the decoupling control strategy, demonstrating that the prototype achieves a maximum normalized power-saving ratio of 42.98% at 10 m/s relative to hover. Furthermore, even when accounting for the 209 g structural weight penalty, the design provides a net 13.22% efficiency improvement over the fixed-incidence concept of the original RflyLW. Leveraging these aerodynamic enhancements, predictive analyses indicate that the UAV can achieve an extended maximum flight endurance of 46.8 min and a practical range of 28.1 km. These results, showing high consistency between numerical predictions and experimental data, offer a robust and highly efficient solution for extending the endurance of UAVs in energy-constrained environments.

**Supplementary Information:**

The online version contains supplementary material available at https://doi.org/10.1038/s41598-026-58543-6.

## Introduction

Quadrotor unmanned aerial vehicles (UAVs) are characterized by their operational versatility, capable of vertical takeoff and landing (VTOL), stable hovering, and precise maneuvering. Such capabilities make them highly valuable for complex missions in urban environments, including aerial photography, infrastructure inspection, and city management^1–3^. However, unlike fixed-wing aircraft that generate passive lift during cruise, quadrotors rely primarily on propeller thrust to maintain altitude. This inherent aerodynamic limitation significantly restricts their endurance and operational range^4,5^. Consequently, enhancing flight endurance within strict size, weight, and power (SWaP) constraints has become a focal point of recent research^6–9^, with bio-inspired morphing wing designs emerging as a promising avenue for aerodynamic efficiency optimization^10^.

Specifically, past research has sought to improve the endurance and flight range of quadrotor UAVs primarily via aerodynamic and structural optimization (including reconfigurable rotor and wing mechanisms^11^, energy system innovation, and hybrid power system design involving novel energy sources such as solar and hydrogen energy. For instance, Xu et al.^12^ proposed an efficient computational aerodynamic optimization method for designing quadrotor propellers based on an advanced Kriging surrogate model implemented in conjunction with a multi-criteria incremental sampling method, which reduced the high-precision computational load by approximately 25%. Lei et al.^13^ explored a comprehensive overview of energy management strategies for quadrotor drones utilizing hybrid power systems, where the optimization of coordinated control across multiple energy sources was investigated in depth, and the intricate challenges posed by nonlinear, strong coupling, and multi-objective constraints were also addressed.

Another prominent approach involves hybrid configurations that combine VTOL capabilities with the passive lift efficiency of fixed wings^6,7,14^. These designs allow for significantly higher cruising speeds. Panigrahi et al.^15^, for example, proposed a dual-tilting-rotor hybrid UAV that transitions between VTOL and fixed-wing flight, successfully extending endurance by reducing the active actuator count. However, the structural footprint and turning radius of such hybrid UAVs often resemble those of conventional fixed-wing aircraft^16^, rendering them ill-suited for operations in confined spaces or obstacle-dense urban airspaces^17,18^, while multi-modal reconfigurable designs face trade-offs between structural complexity and aerodynamic efficiency^19^.

To mitigate these spatial limitations, lifting-wing quadrotors have emerged as a compact hybrid solution^20–26^. These vehicles retain the structural compactness of standard quadrotors while incorporating a wing for passive lift. During hover, they function as conventional drones; during cruise, the fuselage pitches forward, allowing the fixed rotors to provide forward thrust while the wings generate lift, thereby reducing rotor load and power consumption. Despite their advantages, minimizing energy consumption in these designs requires maintaining an optimal lift-to-drag ratio. Since the wing is fixed to the fuselage, the angle of attack (AoA) is strictly coupled with the fuselage pitch angle and flight speed^21,22^.

Previous studies have demonstrated the potential of fixed-incidence lifting wings. Su et al.^27^ achieved an 18.97% power reduction and a 6.9% range increase with a wing mounted at a 30° AoA. Similarly, Wei et al.^28^ reported a 50.14% power reduction at 15 m/s. However, a fundamental limitation persists: maximum efficiency in fixed-wing structures is restricted to a narrow envelope of specific pitch angles and speeds^21,22,29^. In dynamic urban environments requiring frequent speed changes and obstacle avoidance^30–33^, this coupling prevents the UAV from operating at its optimal working point. Therefore, decoupling the AoA from the pitch angle via a variable-incidence mechanism represents a important approach to maximize flight efficiency across the entire flight envelope.

To address these limitations, this paper proposes a novel variable-incidence lifting-wing quadrotor that decouples fuselage attitude from wing aerodynamics. Although the mechanism—comprising a 143 g wing and two 33 g actuators—introduces a 209 g structural weight penalty, it facilitates simultaneous optimization of the wing AoA and fuselage pitch for minimum drag. A prototype based on the DJI Inspire 2^32^ was developed to evaluate aerodynamic characteristics and efficiency gains through Computational Fluid Dynamics (CFD) and flight experiments. The primary contributions of this work are as follows:

- **Flight Validation**: Experimental results support that the variable-incidence structure generates stable lift across a wide range of pitch angles and flight speeds.
- **Optimization Analysis**: Numerical simulations identify that maximum lift is achieved at a fuselage pitch angle of − 20° combined with a wing AoA of 10°–12°.
- **Efficiency Gains**: Flight tests demonstrate a significant reduction in power consumption. Compared to hovering (400.48 W), power consumption during cruising at 10 m/s decreases to 227.77 W, representing a 42.98% reduction. Furthermore, the proposed design achieves a 13.22% higher power reduction efficiency compared to the fixed-incidence concept of the original RflyLW drone at the same speed.
- **Application Viability**: The results indicate that the proposed drone is applicable for long-endurance missions in semi-enclosed or confined environments, such as subway tunnels, mine shafts, and post-disaster urban search-and-rescue scenarios.

## Results and discussion

### Proposed variable-incidence lifting-wing quadrotor design

**Fig. 1:**
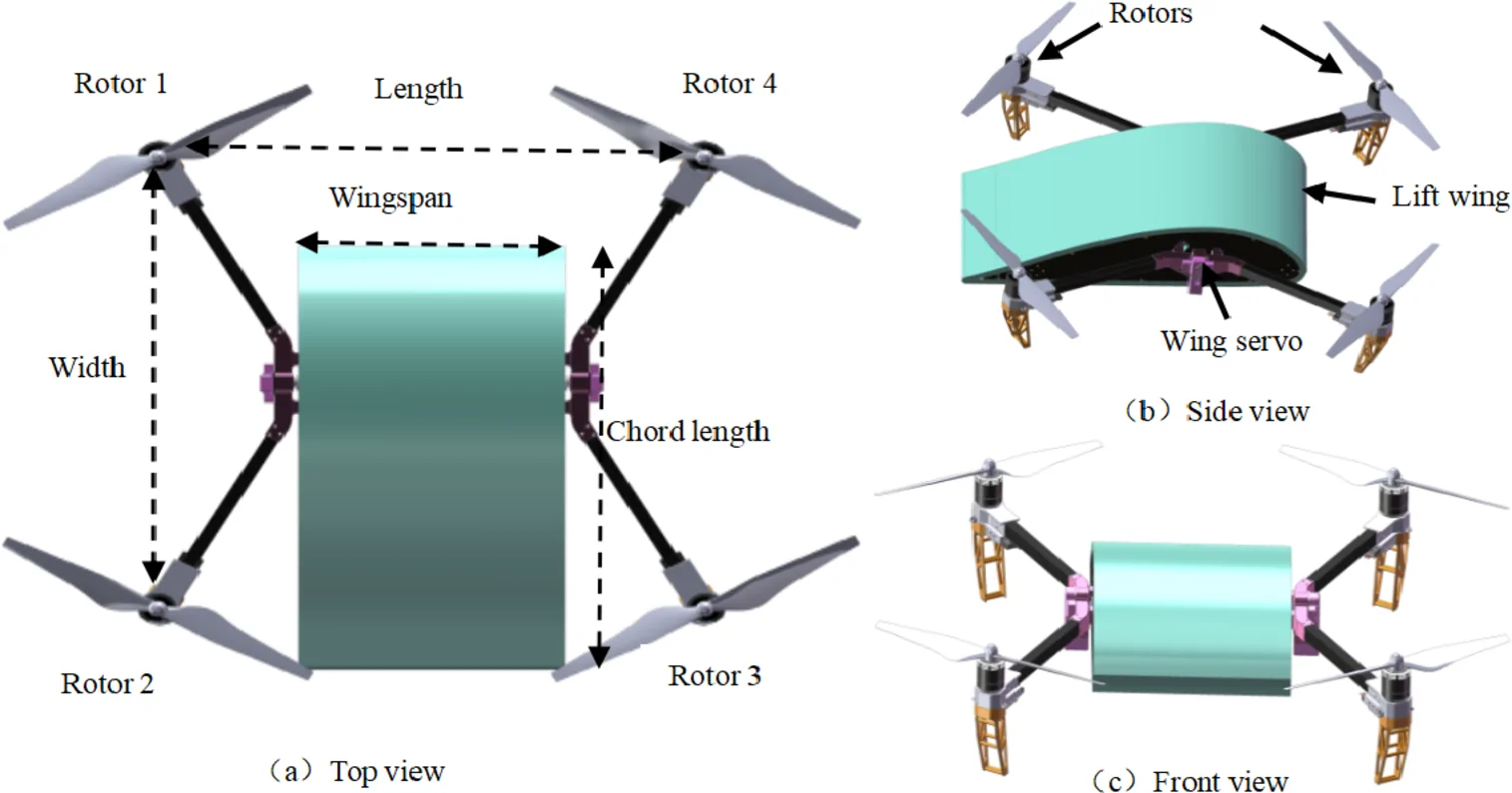
Proposed variable-incidence lifting-wing quadcopter drone design.

The structural configuration of the proposed variable-incidence lifting-wing quadrotor is illustrated in Fig. 1. The design comprises three primary subsystems: a quadrotor airframe, a centrally mounted mono-wing with an adjustable AoA, and a dual-servo actuation mechanism for wing control. Notably, the pair of actuators is symmetrically integrated on both sides of the mono-wing, positioned at one-third of the chord length from the leading edge. To ensure operational reliability and establish a performance baseline comparable to commercial standards, the propulsion system incorporates components from the DJI Inspire 2 platform^34^, specifically utilizing DJI 3512 KV460 brushless DC (BLDC) motors paired with 15-inch DJI 1550 T propellers.

#### Analytical UAV performance analysis

**Fig. 2:**
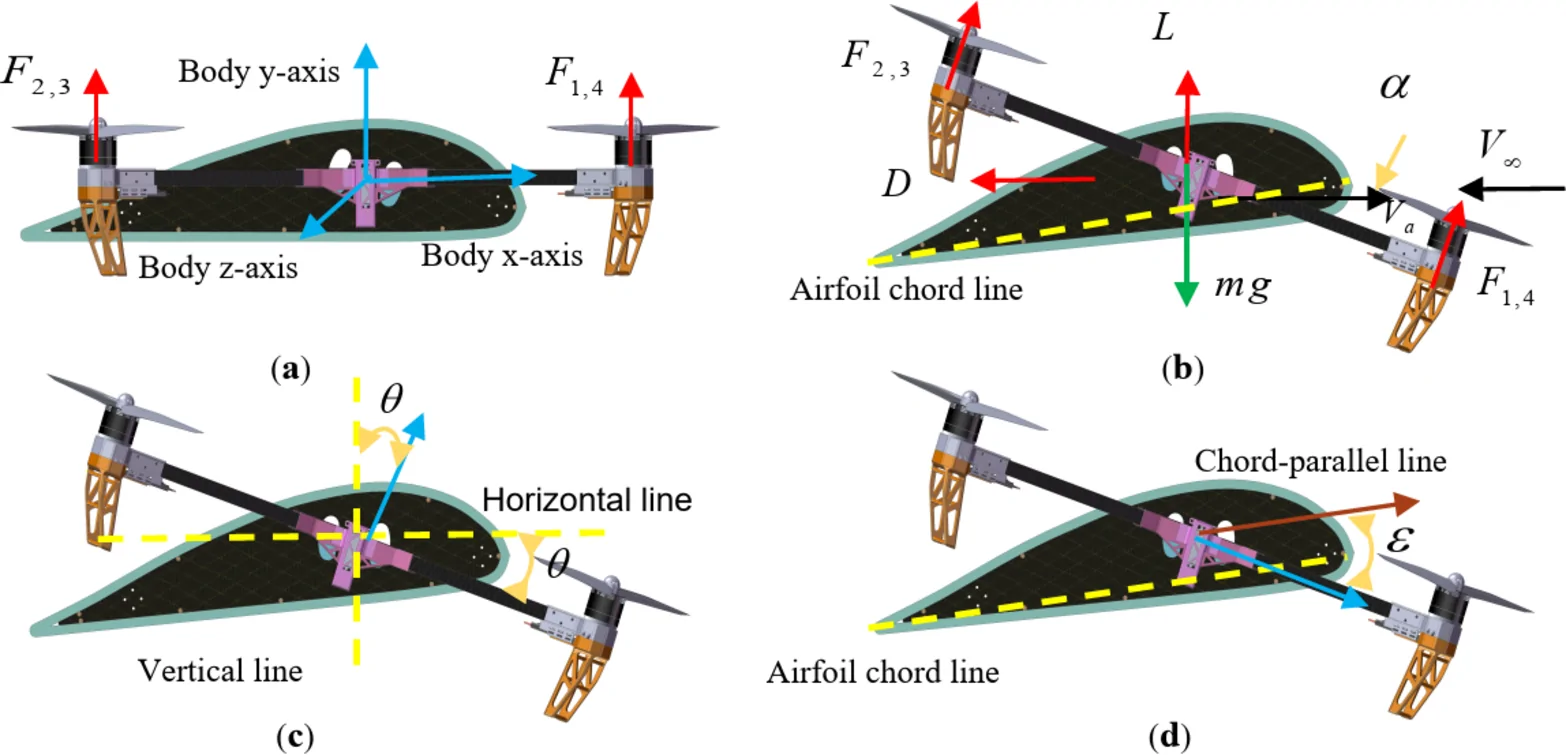
Kinematic and dynamic modeling of the proposed variable-incidence lifting-wing quadrotor. (**a**) Definition of the body coordinate system and thrust vectors during vertical takeoff or hovering. (**b**) Force analysis in steady horizontal forward flight, illustrating the interplay among rotor thrust ($\:{\mathrm{F}}_{\mathrm{i}}$), wing lift (* L*), wing drag (* D*), gravity ($\:\mathrm{m}\mathrm{g}$), flight velocity ($\:{\mathrm{V}}_{\mathrm{a}}$) and freestream velocity ($\:{\mathrm{V}}_{{\infty\:}}$). (**c**) Definition of the fuselage pitch angle ($\:\theta\:$) relative to the horizontal plane. (**d**) Definition of the variable wing incidence angle (ε), representing the geometric angle between the airfoil chord line and the fuselage longitudinal axis (body x-axis).

The individual forces associated with the proposed quadrotor UAV are comprehensively defined in Fig. 2.

As illustrated in Fig. 2(a) and 2(c), in the body coordinate system, the quadrotor fuselage is pitched forward by an angle $\:\theta\:$ during forward flight. Simultaneously, as detailed in Fig. 2(d), the lifting wing is deflected by an incidence angle *ε* relative to the body x-axis. This mechanical deflection, coupled with the fuselage pitch attitude, establishes the effective aerodynamic Angle of Attack (AoA) $\:\alpha\:$ relative to the local airflow velocity $\:{\mathrm{V}}_{{\infty\:}}$ induced by the drone’s horizontal flight speed $\:{\mathrm{V}}_{\mathrm{a}}$. Consequently, the wing generates a passive lift force * L*. The total thrust produced by the four rotors is denoted as $\:\sum\:_{\mathrm{i}=1}^{4}{F}_{i}$. The aerodynamic lift generated by the wing can be expressed as:

1 $$\:L=\frac{1}{2}{\uprho\:}{\mathrm{V}}^{2}\mathrm{S}{C}_{L\left(\alpha\:\right)}$$

Where $\:\rho$ denotes the air density, * S* represents the wing planform area, and $\:{C}_{L\left(\alpha\:\right)}$ is the lift coefficient of the wing, which is a function of $\:\alpha\:$.

Under steady-state, low-speed horizontal cruise conditions, the force equilibrium along the body z-axis can (perpendicular to the tilted fuselage, as seen in Fig. 2b) can be approximated by balancing the thrust, the normal component of the wing lift, and the normal component of gravity:

2 $$\:\sum\:_{\mathrm{i}=1}^{4}{\mathrm{F}}_{\mathrm{i}}+\mathrm{L}\mathrm{c}\mathrm{o}\mathrm{s}{\uptheta\:}\approx\:\mathrm{m}\mathrm{g}\mathrm{c}\mathrm{o}\mathrm{s}{\uptheta\:}$$

Rearranging this equation yields the required rotor thrust:

3 $$\:\sum\:_{\mathrm{i}=1}^{4}{\mathrm{F}}_{\mathrm{i}}\approx\:\mathrm{m}\mathrm{g}\mathrm{c}\mathrm{o}\mathrm{s}{\uptheta\:}-\mathrm{L}\mathrm{c}\mathrm{o}\mathrm{s}{\uptheta\:}$$

Drawing upon momentum theory for rotorcraft^35^, the flight power consumption * P* of the lifting-wing quadrotor can be modeled as a function of the required thrust:

4 $$\:P={V}_{B}I\propto\:\mathrm{n}\sqrt{\frac{(\mathrm{m}\mathrm{g}\mathrm{c}\mathrm{o}\mathrm{s}{\uptheta\:}-\mathrm{L}\mathrm{c}\mathrm{o}\mathrm{s}{\upalpha\:})}{2{\uprho\:}\mathrm{A}{\mathrm{n}}^{3}}}$$

Here, $\:{V}_{B}$ represents the battery voltage, * I* is the battery discharge current, $\:\mathrm{n}$ is the number of rotors, and * A* is the area of each rotor disk.

Assuming *θ* and α are small angles, combining Eqs. (1)–(4) reveals the sensitivity of power consumption * P* to key aerodynamic parameters:

- P decreases as the wing area $\:\mathrm{S}$ and lift coefficient $\:{C}_{L\left(\alpha\:\right)}$ increase;
- P decreases as the flight velocity $\:{V}_{\mathrm{a}}$ increases (within the low-speed regime) due to enhanced passive lift generation;
- P decreases as the magnitude of the pitch angle $\:\theta$ reduces (approaching horizontal).

This analysis highlights an inherent trade-off in conventional fixed-wing hybrid quadrotors. While reducing the pitch angle $\:\theta$ lowers the vertical thrust requirement and thus power, it simultaneously reduces the horizontal thrust component required to maintain the forward velocity $\:{V}_{\mathrm{a}}$. Therefore, decoupling the fuselage attitude from the wing AoA—specifically the ability to optimize wing AoA $\:{\upalpha\:}$ via the variable-incidence mechanism (angle ε) independently of $\:\theta$—becomes the effective method for minimizing power consumption. Consequently, the wing design plays a pivotal role in enhancing the energy efficiency of lifting-wing quadrotors.

#### Airfoil selection

The GOE 384 airfoil^36^ was adopted for the lifting surfaces. While our variable-incidence prototype and the RflyLW drone^8,21,25^ share a comparable total wing area (approx. 0.16 m²), they represent divergent aerodynamic paradigms (Table 1). Our design utilizes an ultra-low aspect ratio (AR ≈ 0.61) configuration integrated with a dynamic incidence mechanism (0°–30°) to enable active lift modulation and stall control across an expanded flight envelope. Conversely, the RflyLW employs a high-aspect-ratio (AR ≈ 5.53) geometry with a fixed 34° incidence, prioritizing conventional aerodynamic efficiency. The increased gross mass and upgraded propulsion system of the proposed UAV reflect a necessary structural-aerodynamic trade-off to facilitate these advanced maneuverability capabilities.

**Table 1:** Specific parameters of the proposed and RflyLW drone^8,21,25^.

Parameter	Proposed Prototype	RflyLW Drone [8, 21, 25]
Gross mass (kg)	3.40	1.92
Dimensions (L×W×H, mm)	750 × 650 × 350	750 × 94 × 289
Chord length (mm)	510	170
Wingspan (mm)	310	940
Aspect ratio	0.61	5.53
Incidence angle (°)	0–30	34 (fixed)
Battery pack	6S2P Samsung INR21700-50E [41], 10 Ah	—
Propulsion system	DJI 3512 Motor/DJI 1550 T Propeller [34]	—

### Aerodynamic characteristics of the isolated lifting wing

The aerodynamic characteristics of the isolated GOE 384 lifting wing were analyzed independently of the fuselage and rotors to establish a baseline for performance evaluation.

Firstly, the variation of aerodynamic forces with the AoA was investigated. Table 2 summarizes the computed lift * L*, drag * D*, and their respective coefficients $\:{C}_{l}$, and $\:{C}_{d}$ under a constant cruising velocity of 12 m/s. The results indicate a positive correlation between lift and AoA: as the AoA increases from 4° to 12°, the generated lift rises significantly from 5.08 N to 9.74 N. Conversely, the lift-to-drag ratio $\:{C}_{l}/{C}_{d}$ exhibits a declining trend, decreasing from 4.59 to 3.97. This reduction is attributed to the rapid accumulation of drag, particularly the induced drag inherent to the low aspect ratio of the wing, which outpaces the gain in lift at higher AoAs.

**Table 2:** Computed aerodynamic characteristics of the isolated GOE 384 lifting wing under a horizontal airflow velocity of 12 m/s.

AoA (°)	Lift (*N*)	Drag (*N*)	$\:{C}_{l}$	$\:{C}_{d}$	Lift-to-drag Ratio
4	5.08	1.107	0.28	0.06	4.59
6	6.17	1.35	0.34	0.07	4.57
8	7.00	1.63	0.39	0.09	4.30
10	8.49	2.01	0.47	0.11	4.22
12	9.74	2.45	0.54	0.14	3.97

**Fig. 3:**
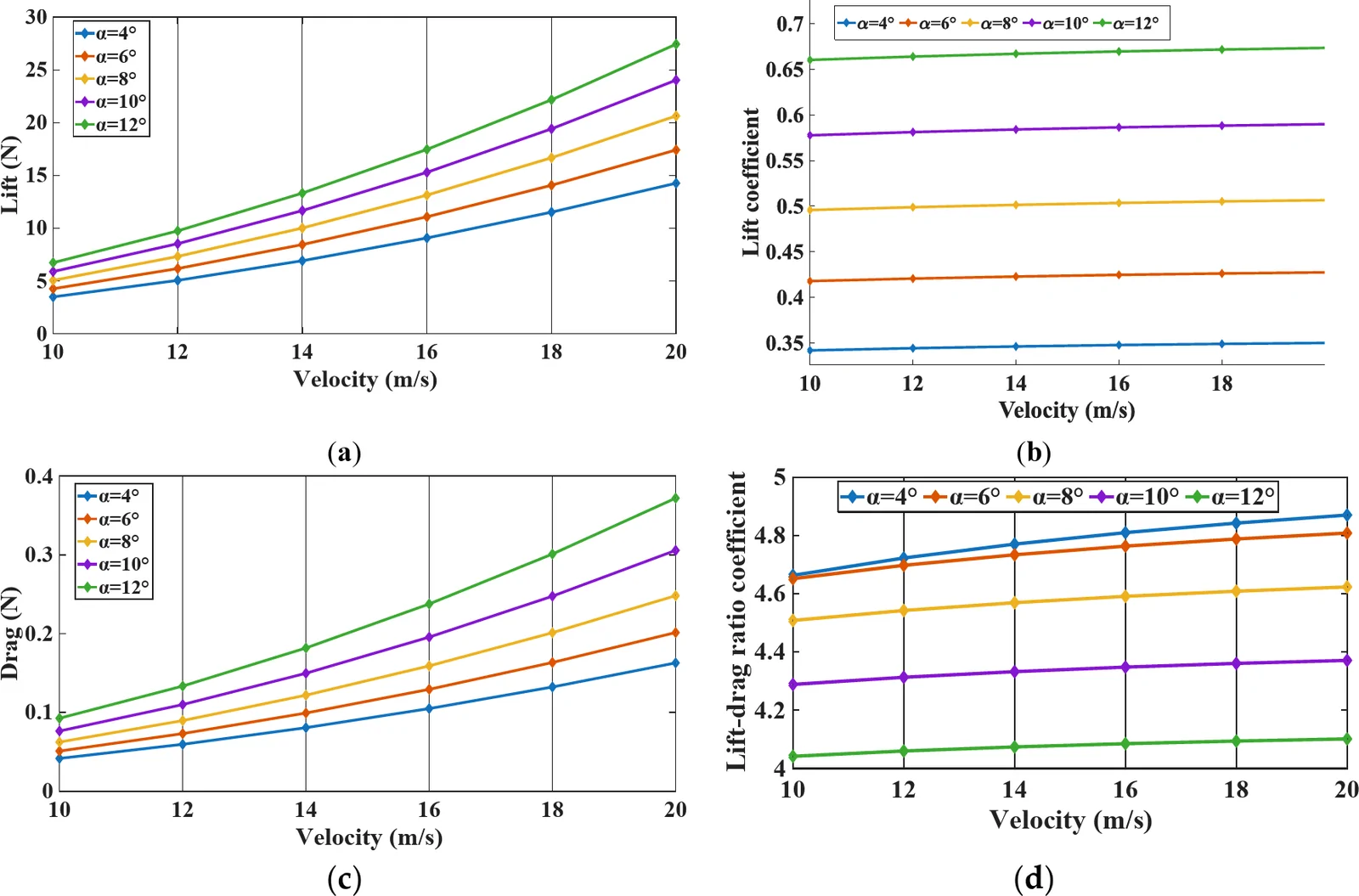
Computed aerodynamic characteristics of the GOE 384 lifting wing with the parameters listed in Table 1 as a function of horizontal airflow velocity at different AoA ($\:{\upalpha\:}$) values: (**a**) lift; (**b**) lift coefficient; (**c**) drag; (**d**) lift-to-drag ratio.

Subsequently, the coupled effects of flight velocity and AoA were evaluated. Figure 3 illustrates the aerodynamic performance maps across a velocity range of 10–20 m/s.

- **Forces and Coefficients (**Fig. 3a-c**)**: It is observed that the lift force, lift coefficient, and drag force increase monotonically with both increasing velocity and AoA.
- **Aerodynamic Efficiency (**Fig. 3d**)**: The lift-to-drag ratio demonstrates a distinct behavior; it minimizes at 12 m/s and generally decreases as the AoA increases, highlighting the trade-off between lift generation and drag penalty.

**Fig. 4:**
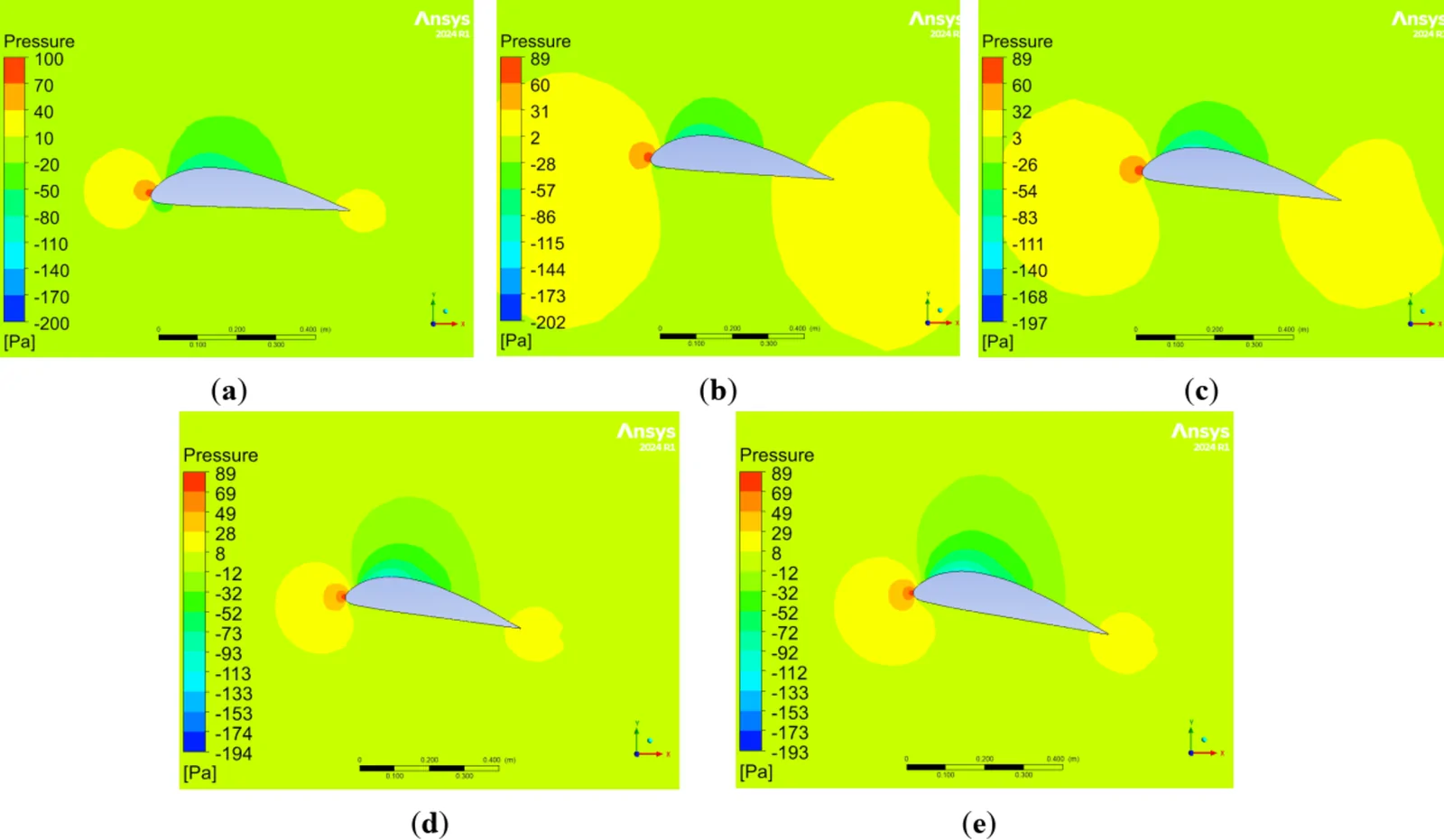
Representative contour plots of the 2D pressure distributions computed around the midpoint of the GOE 384 lifting wing at different AoA values: (**a**) 4°; (**b**) 6°; (**c**) 8°; (**d**) 10°; (**e**) 12°.

Finally, the flow mechanism governing lift generation was visualized via pressure distributions. Figure 4 presents the 2D pressure contours at the mid-span section of the wing at 12 m/s.

- A distinct low-pressure zone (suction side) develops on the upper surface, while a relatively high-pressure zone (pressure side) forms on the lower surface. The pressure differential between these surfaces generates the aerodynamic lift.
- As the AoA increases from 4° to 12°, the suction peak on the upper surface intensifies, and the low-pressure region expands significantly towards the trailing edge. Simultaneously, the stagnation region at the leading edge shifts, further contributing to the increased pressure differential and total lift.

### Aerodynamic characteristics of the proposed drone in forward flight mode with respect to pitch angle

**Fig. 5:**
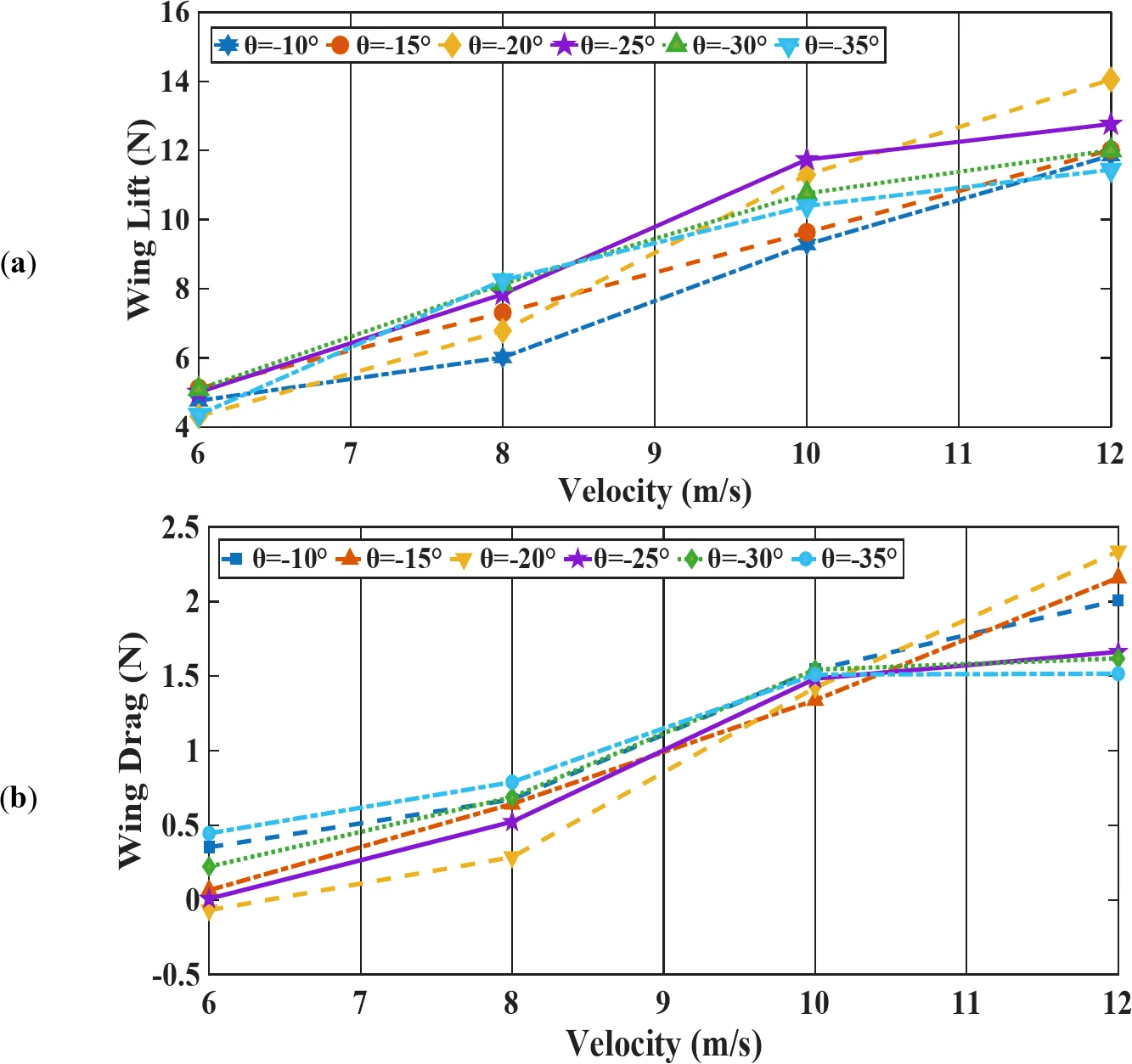
Computed aerodynamic characteristics of the proposed drone in forward flight mode as a function of horizontal airflow velocity at *α* = 10° and different drone pitch angles (*θ*): (**a**) lift; (**b**) drag.

The coupled effects of forward flight velocity and fuselage pitch angle $\:\theta\:$ on the aerodynamic performance were investigated, where the complex aerodynamic interference between rotor wake and wing surface plays a dominant role in the overall flow field characteristics^37^. In these simulations, the wing AoA $\:\alpha\:$ was actively maintained at a fixed value of 10$\:^\circ\:$ to isolate the influence of the fuselage attitude. Figure 5 presents the computed lift and drag characteristics.

As illustrated in the plots, both lift and drag forces exhibit a positive, monotonic correlation with flight velocity across all pitch angles, a behavior consistent with standard aerodynamic theory where dynamic pressure increases with the square of the velocity. However, the relationship between aerodynamic forces and the pitch angle $\:\theta\:$ is notably non-linear, revealing strong aerodynamic interference effects between the rotors and the wing. Specifically, the lifting wing achieves maximum aerodynamic performance at an optimal pitch angle of $\:\theta\:$ = −20$\:^\circ\:$; at a cruising speed of 12 m/s, the peak lift reaches 14.05 N. The system exhibits significant performance sensitivity, where deviating from this optimal angle results in a noticeable reduction in lift—for instance, at $\:\theta\:$ = −20$\:^\circ\:$, the lift decreases to 12.76 N under identical conditions. Regarding the drag forces, a corresponding value of 2.36 N is recorded for the optimal $\:\theta\:$ = −20$\:^\circ\:$ configuration at the same cruising speed.

**Fig. 6:**
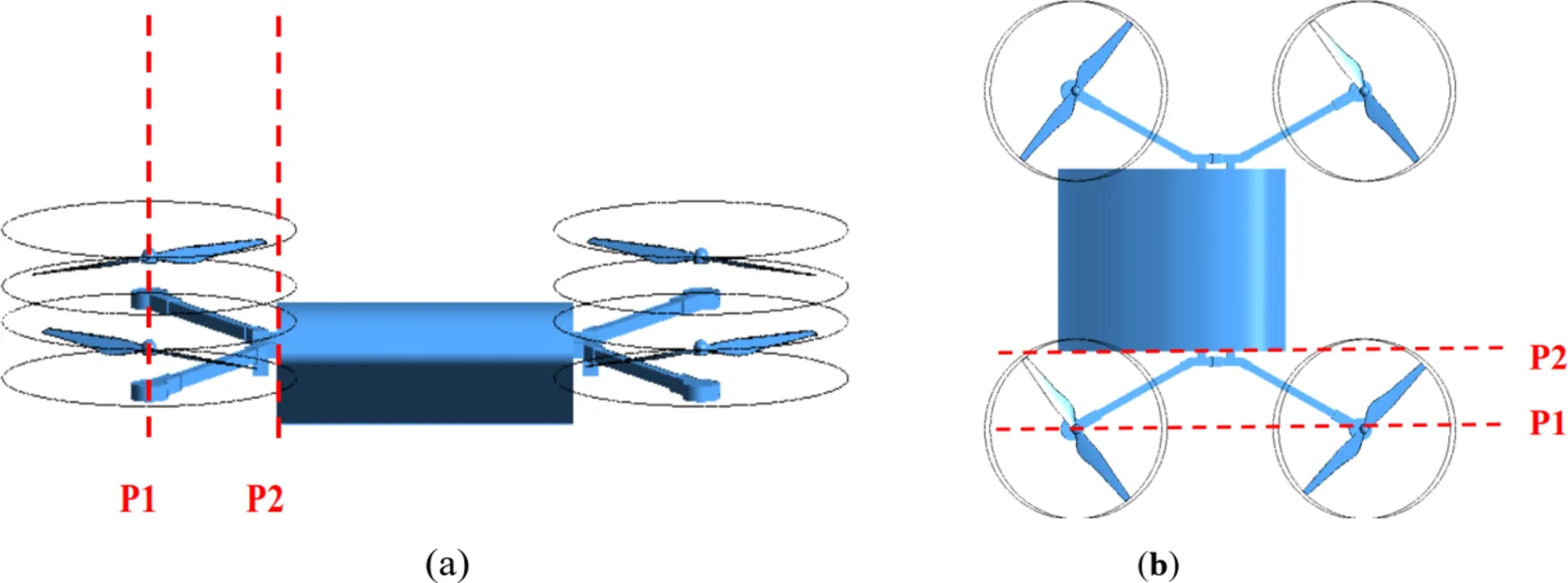
Schematic representation defining the locations of the longitudinal extraction planes used for the 2D flow field analysis. The frontal oblique view (**a**) and the top-down view (**b**) clearly illustrate two critical sections: the rotor-axis section (**P1**), which passes directly through the rotation centers of the tandem rotors, and the primary aerodynamic interference section (**P2**), which is located adjacent to the left wing mounting junction.

**Fig. 7:**
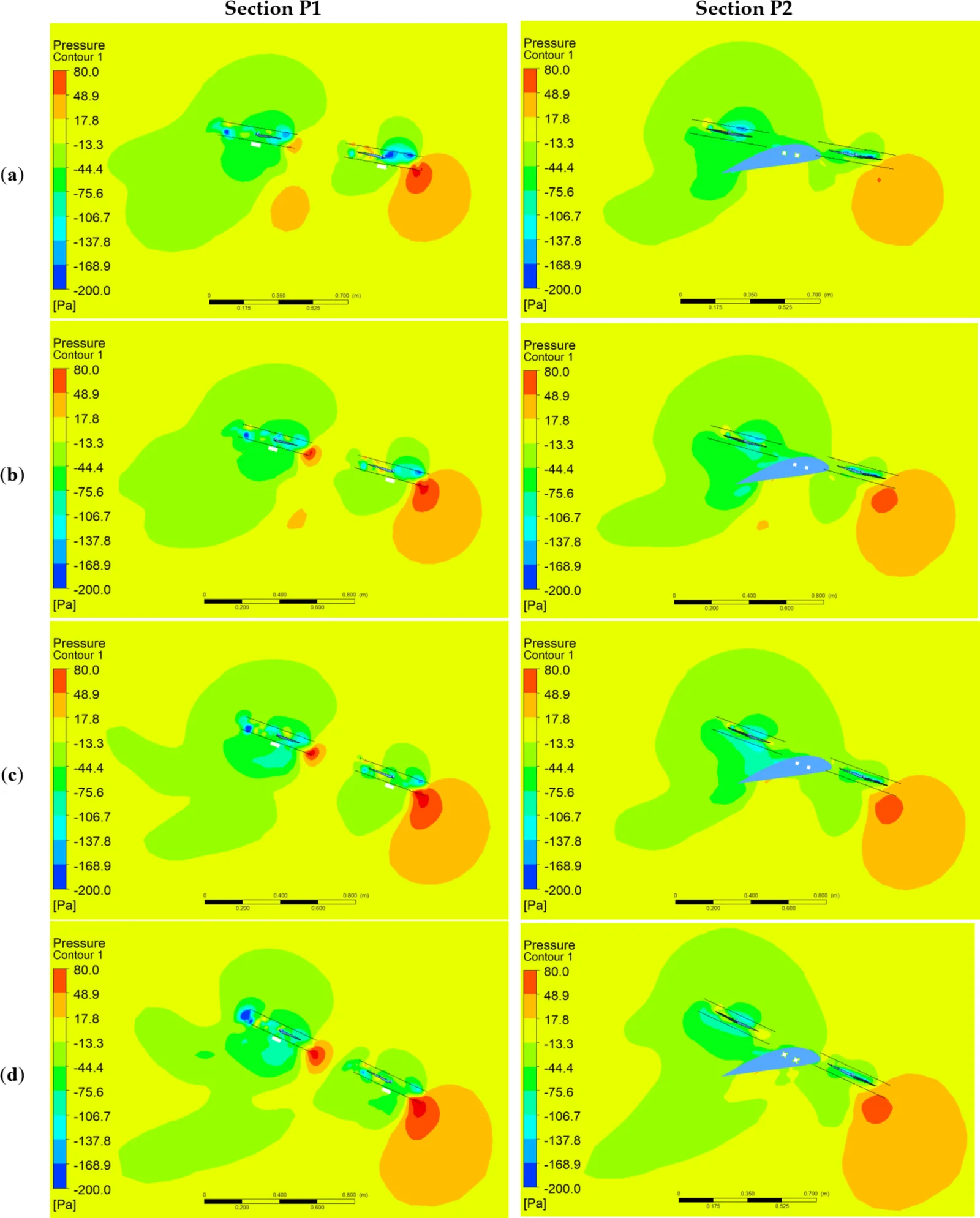

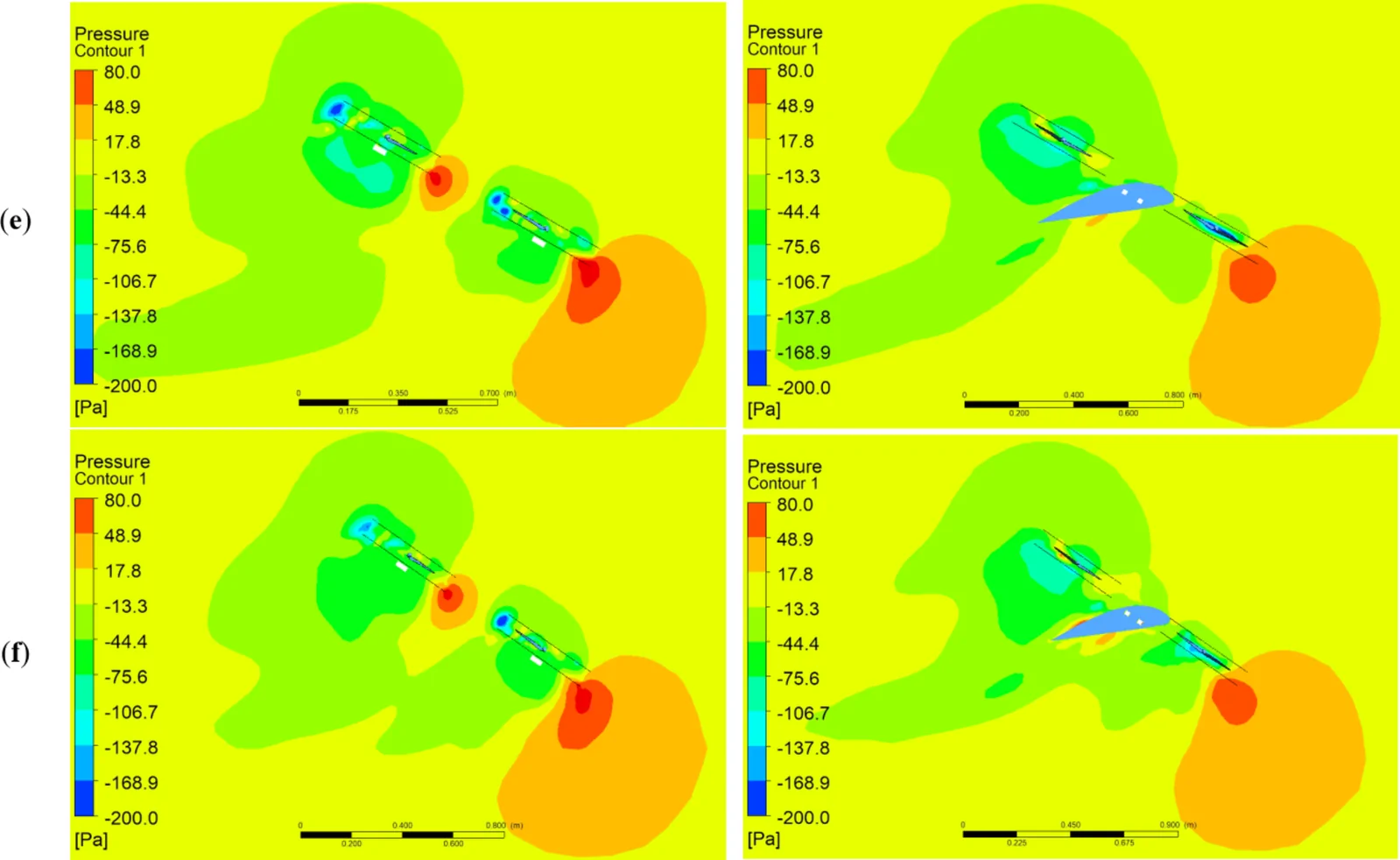
Computed static pressure contour plots illustrating aerodynamic interference in forward flight mode at a fixed wing angle of attack $\:{\upalpha\:}=10$° across varying fuselage pitch angles *θ*. Section P1 represents the longitudinal plane passing through Rotor 3 and Rotor 4, while Section P2 corresponds to the left mounting surface. The corresponding pitch angles from top to bottom are: (**a**) − 10°; (**b**) − 15°; (**c**) − 20°; (**d**) − 25°; (**e**) − 30°; (**f**) − 35°.

These macroscopic force variations suggest that while the variable-incidence mechanism maintains the wing’s local wing AoA $\:\alpha\:$, the global airflow environment is heavily dependent on the fuselage pitch angle. This macroscopic behavior is consistent with the well-documented rotor-on-wing interference phenomena observed in compound-wing UAVs during forward flight^29,38^. To physically elucidate how this rotor-wing interaction alters the lift generation, cross-sectional pressure contour plots were extracted at two critical longitudinal planes, as defined in Fig. 6: the rotor-axis section (P1) and the primary aerodynamic interference section (P2). Subsequently, Fig. 7 illustrates the 2D pressure distributions across two these sections at a fixed wing AoA $\:\alpha\:$ of 10° under various pitch angles.

These contours demonstrate the direct coupling between the rotors’ dynamic pressure fields of the rotors and the lifting wing. Focusing on the interference section (P2), at moderate pitch angles corresponding to peak performance (e.g., *θ*=−20°), the negative pressure region (particularly the prominent suction peak) on the upper surface of the wing remains relatively intact and distinct from the rotor wake, indicating efficient lift generation. However, as the magnitude of the pitch angle increases (e.g., *θ*=−30°), severe aerodynamic interference emerges. The intense low-pressure zone generated by the forward rotor expands downward and merges with the wing’s leading-edge boundary layer. This “swallowing” effect severely disrupts the local pressure gradient, leading to premature flow separation on the wing. Simultaneously, observing the rotor-axis section (P1), the rear rotor becomes fully immersed in the high-pressure downwash trailing from the wing, which significantly degrades its operating environment and contributes to the overall drag increase. Consequently, *θ*=−20° represents an optimal aerodynamic trade-off configuration, where the wing and rotors coexist with minimal destructive interference.

**Fig. 8:**
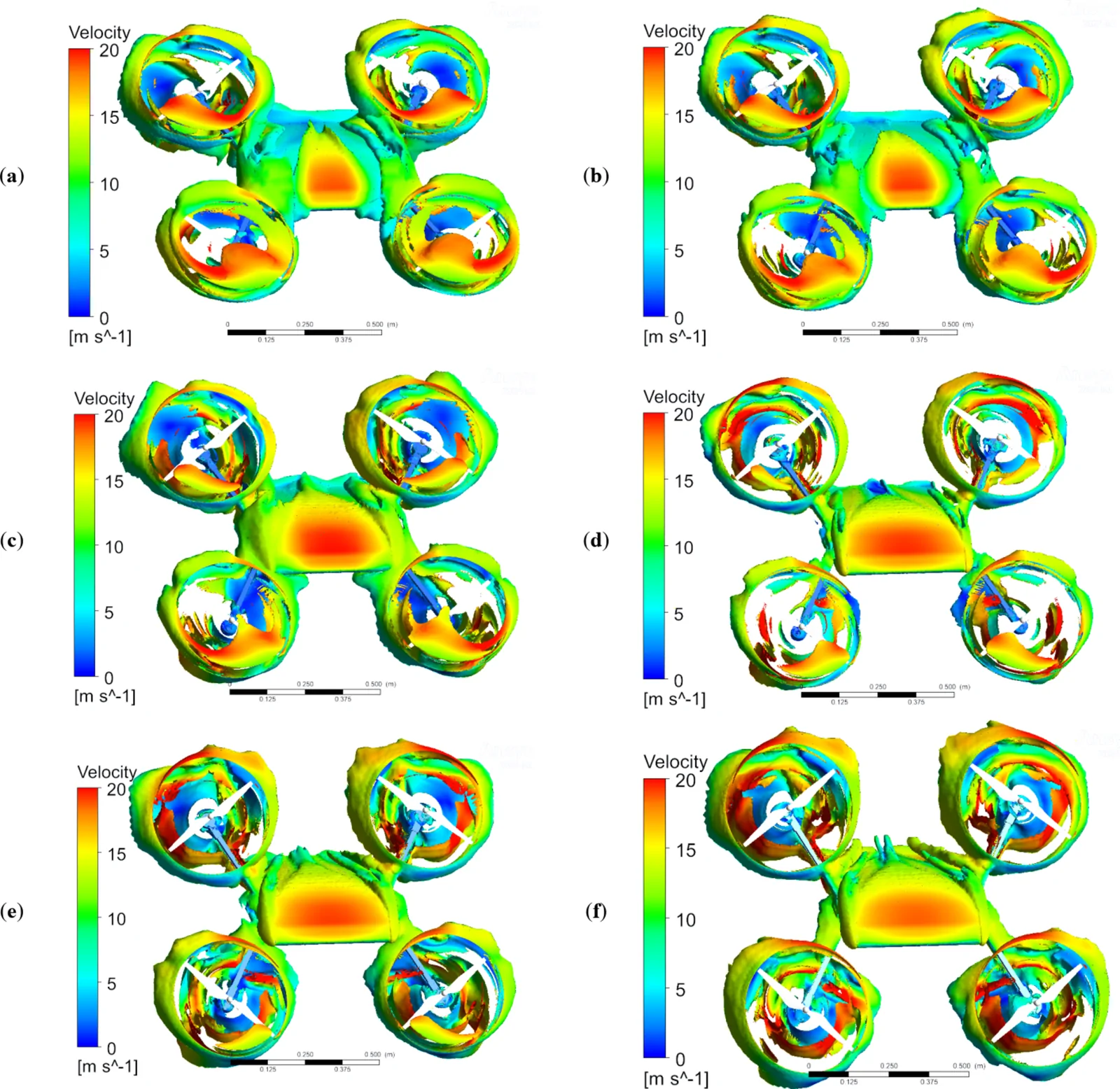
Computed 3D vortex-velocity contour plots at the top (inflow) of the proposed drone in forward flight mode under a horizontal airflow velocity of 10 m/s at *α* = 10° and different values of *θ.* Vortex structures are identified by a vorticity magnitude threshold of 0.0005s^−1^, colored by velocity magnitude: −10°; (**b**) − 15°; (**c**) − 20°; (**d**) − 25°; (**e**) − 30°; (**f**) − 35°.

This severe disruption in the surface pressure distribution is fundamentally driven by the behavior of the spatial vortex structures. To visualize the global wake dynamics causing these aforementioned pressure shifts, the 3-D vortex topology was subsequently analyzed. Figure 8 presents the vortex structures (identified by a vorticity magnitude threshold of 0.0005 s^− 1^ colored by velocity magnitude) under a flight velocity of 10 m/s and a fixed wing AoA $\:\alpha\:$ of 10°, across various pitch angles *θ*。.

As illustrated in Fig. 8(a), at a shallow pitch angle *θ*=−10, the rotor wake directly impinges upon the upper surface of the lifting wing. This strong aerodynamic interference suppresses the development of the flow field, resulting in a constrained high-velocity suction region. However, as the drone tilts further forward, decreasing *θ* from − 15 to − 25°, the rotor wake is convected downstream, gradually clearing the wing surface. Consequently, the high-velocity vortex region on the wing’s upper surface expands significantly (Figs. 8b-d), indicating the re-establishment of a strong suction peak. This flow attachment behavior corroborates the intact pressure profiles seen at optimal angles and correlates directly with the peak lift performance. Finally, with further inclination to − 30° and − 35°(Figs. 8e-f), the effective aerodynamic cross-section changes, and the coherent suction area diminishes, corresponding to the severe boundary layer disruption previously observed in the pressure contours and the resulting decline in macroscopic lift force.

### Aerodynamic characteristics in the forward flight mode with respect to AoA

**Fig. 9:**
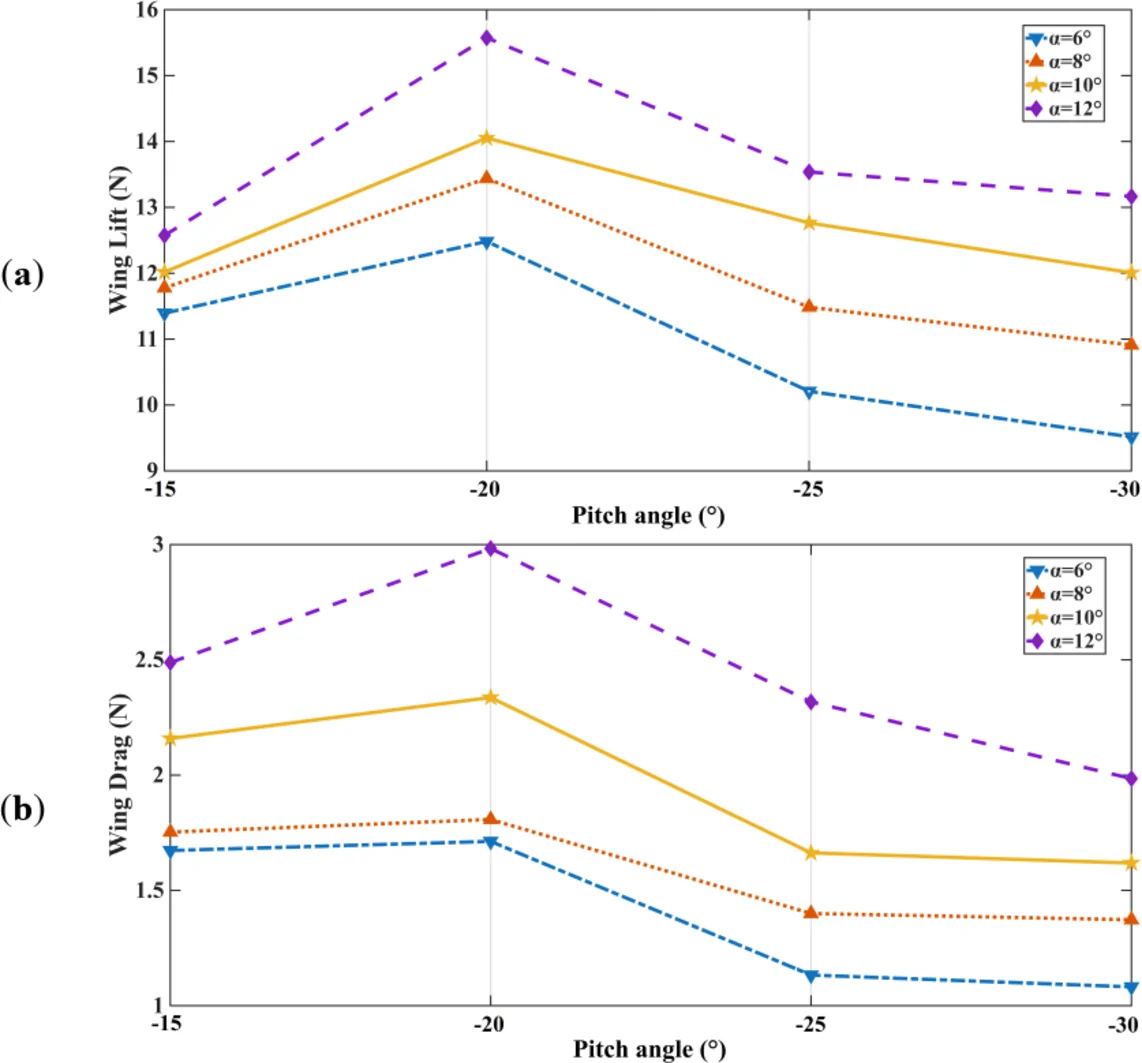
Computed aerodynamic characteristics of the proposed drone as a function of *θ* under a horizontal airflow velocity of 10 m/s at different values of *α*: (**a**) lift; (**b**) drag.

The coupled aerodynamic effects of the fuselage pitch angle $\:\theta\:$ and the wing AoA $\:\alpha\:$ were investigated at a constant cruising speed of 10 m/s, with the resulting variations in lift and drag forces summarized in Fig. 9. Observations indicate that the aerodynamic forces exhibit a positive, monotonic correlation with the wing AoA $\:\alpha\:$. Specifically, for any fixed pitch angle, increasing wing AoA $\:\alpha\:$ from 6° to 12° results in a linear increase in both lift and drag. This linear response supports that the variable-incidence mechanism effectively controls the local flow conditions over the wing, independent of the fuselage attitude. In contrast, the influence of the fuselage pitch angle is distinctly non-linear. As the magnitude of forward tilt increases from 15° to 30°, the forces follow a concave trend: both lift and drag initially rise to reach a global maximum at $\:\theta\:$ = −20°, yielding a peak lift of 15.57 N and drag of 2.98 N at $\:\alpha\:$ = 12°, before degrading significantly at steeper angles ($\:\theta\:$ = −25° and − 30°). This sharp decline indicates that excessive forward tilt introduces detrimental wake interference or flow separation, consistent with the qualitative analysis in Sect. 2.3. Collectively, these results demonstrate that while the wing AoA $\:\alpha\:$ provides linear control authority over lift generation, the fuselage pitch angle $\:\theta\:$ dictates the global flow environment, identifying $\:\theta\:$ = −20° as the optimal aerodynamic operating point for this specific UAV configuration.

**Fig. 10:**
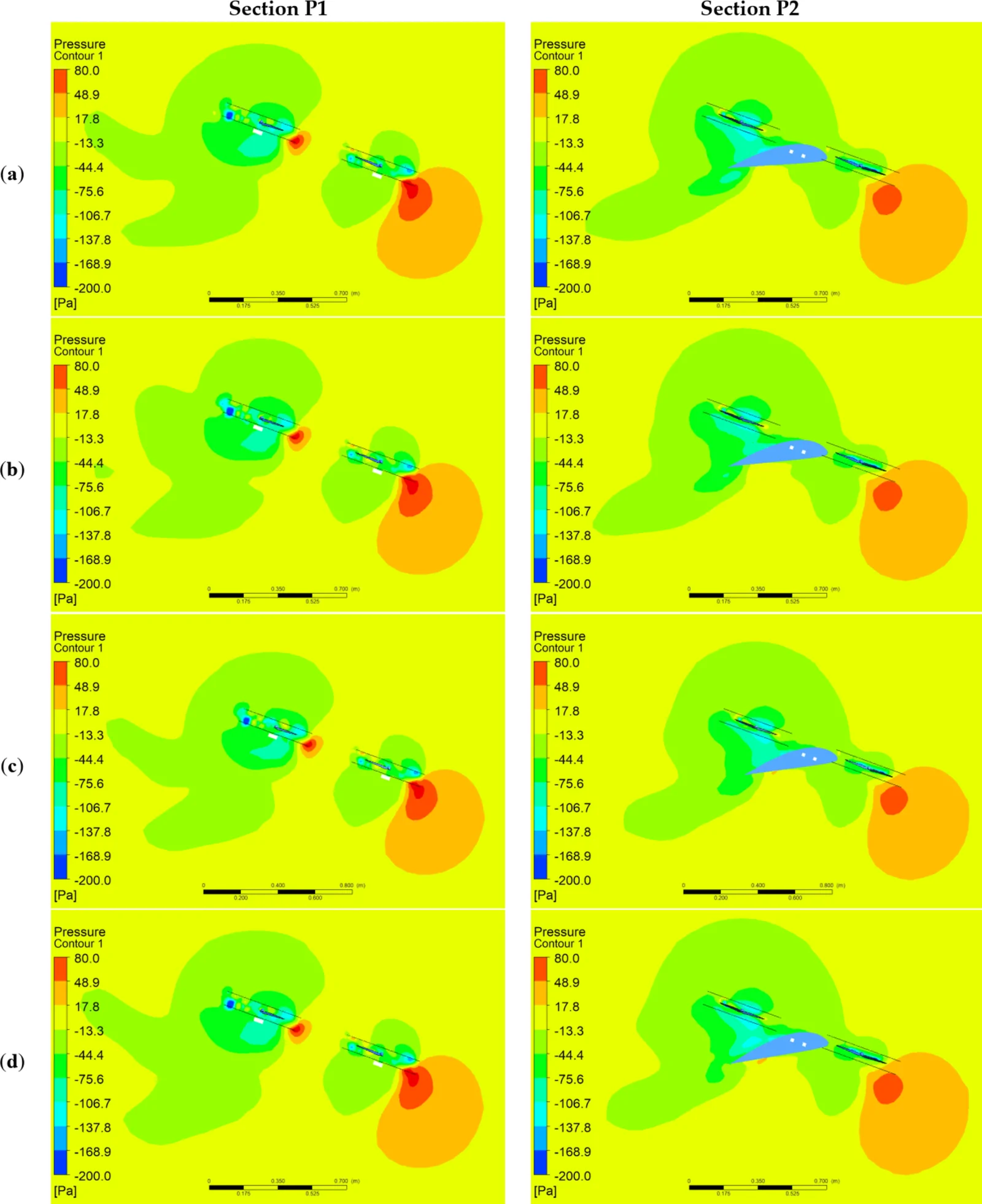
Computed static pressure contour plots illustrating the flow field evolution in forward flight mode at the optimal fuselage pitch angle ($\:\theta\:$ = −20°) across varying wing angles of attack $\:{\upalpha\:}$. Section P1 represents the longitudinal plane passing through Rotor 3 and Rotor 4, while Section P2 corresponds to the left mounting surface. The corresponding AoAs from top to bottom are: (**a**) 6; (**b**) 8; (**c**) 10; (**d**) 12.

To physically validate the mechanism behind this linear, stall-free lift augmentation at the optimal pitch angle, cross-sectional pressure distributions were examined. Figure 10 presents the static pressure contours across the rotor-axis section (P1) and the interference section (P2), locations of which were previously defined in Fig. 6, at a fixed $\:\theta\:$ = −20° under varying AoAs. Focusing on the interference section (P2), a clear and healthy aerodynamic progression is observed. As the wing AoA $\:\alpha\:$ increases from 6 to 12 (Figs. 10a-d), the negative pressure zone on the wing’s upper surface progressively expands and intensifies, which directly translates to the linear lift increase. Crucially, unlike the severe interference observed at extreme pitch angles in Sect. 2.3, the forward rotor’s wake at $\:\theta\:$ = −20° maintains a safe clearance from the wing. The wing’s leading-edge suction peak remains intact and isolated from the rotor’s low-pressure field, even at the highest tested wing AoA $\:\alpha\:$ of 12°.

**Fig. 11:**
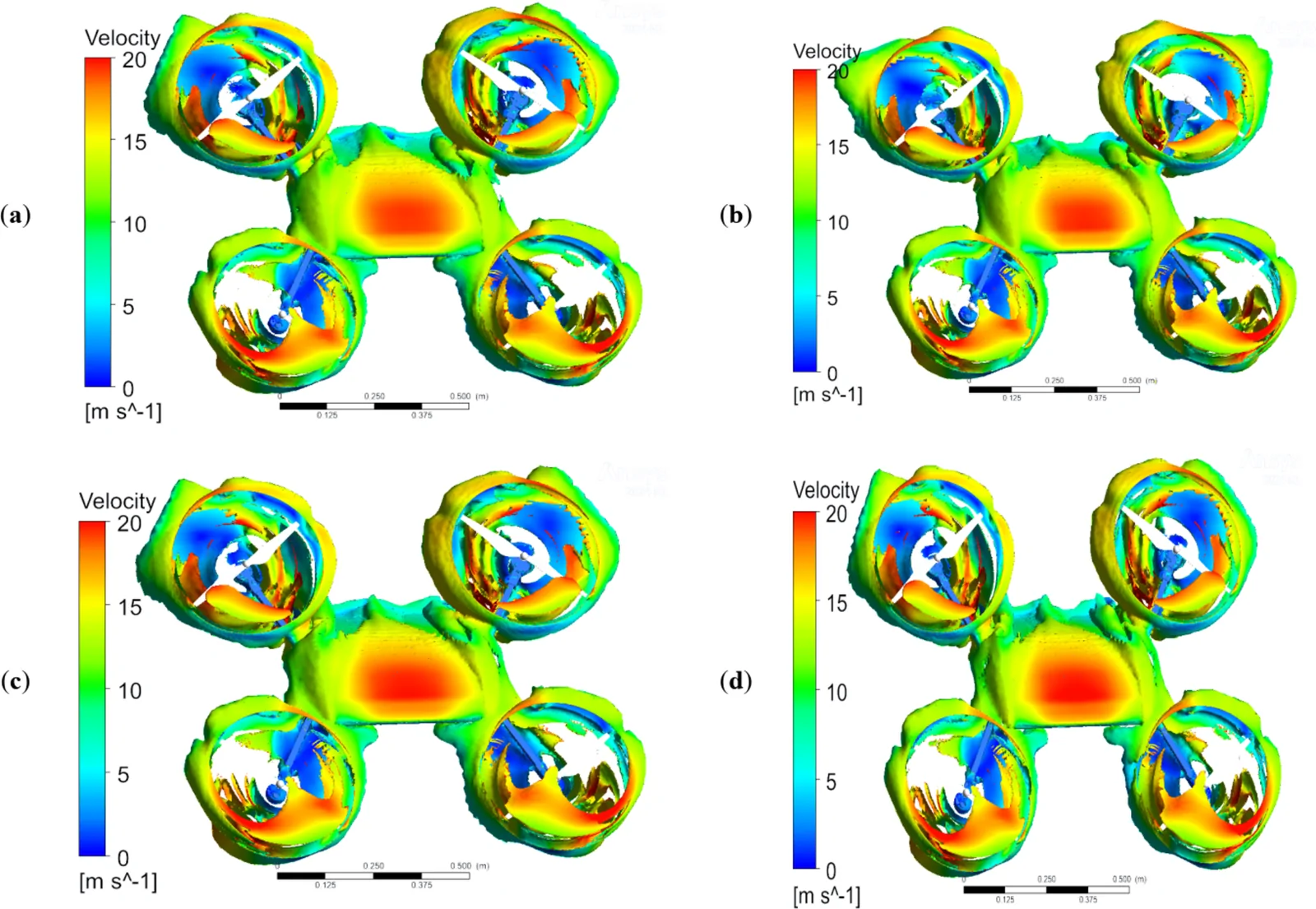
Computed 3D vortex-velocity contour plots (top/inflow perspective) in forward flight (*V* = 10 m/s, $\:\theta$ = −20°) at varying wing AoAs $\:{\alpha}$. Vortex structures are identified by a vorticity magnitude threshold of 0.0005s^−1^, colored by velocity magnitude: (**a**) 6°; (**b**) 8°; (**c**) 10°; (**d**) 12°. Note the progressive expansion of the high-velocity suction region on the wing surface.

The fundamental flow physics governing this healthy pressure distribution and linear lift increase are further elucidated through an analysis of the three-dimensional vortex topology. Figure 11 presents the vortex structures under the optimal pitch angle condition ($\:\theta\:$ = −20°) at a cruising velocity of 10 m/s across varying wing AoAs (*α* = 6, 8, 10, and 12). The visualization reveals a clear evolution of the flow field on the wing’s upper surface: as $\:\alpha\:$ increases, the high-velocity vortex region (indicated in red) expands progressively in both spanwise and chordwise directions. Physically, this expansion corresponds directly to the intensification of the suction peak observed in the pressure contours (Fig. 10). Furthermore, consistent with the pressure data, even at the highest tested wing AoA $\:\alpha\:$ of 12, the vortex structure remains coherent and intact, showing no signs of breakdown or gross flow separation. This stability indicates that at the optimal pitch angle of $\:\theta\:$ = −20°, destructive rotor wake interference is minimized, thereby allowing the wing to operate efficiently within its linear lift region.

### Proposed UAV flight verification

A prototype of the variable-incidence lifting-wing quadrotor was fabricated based on the parameters in Table 1, providing experimental validation for the numerical simulations in Sect. 2.4. This platform features a custom-developed flight controller for the variable-incidence mechanism and a high-capacity 6 S lithium-ion battery (25.2 V, 10 Ah, 50 A maximum discharge) to ensure a consistent power supply.

A specific control law was implemented to decouple the wing angle of attack (AoA) from the fuselage pitch angle $\:\theta\:$, aiming to quantify the aerodynamic benefits of the lifting wing. Although the theoretical effective AoA involves the induced downwash angle $\:{\alpha\:}_{ind\:}$(as defined in Eq. (4)), measuring this term in real time inevitably necessitates complex sensor arrays. Therefore, for the sake of engineering verification simplification, the dynamic $\:{\alpha\:}_{ind}$ term is omitted. The applied control strategy directly compensates for the measurable pitch angle $\:\theta\:$, yielding the approximated governing equation:

5 $$\:{\alpha\:}_{eff\:}=\epsilon\:+{\epsilon\:}_{0\:}-(K-1)\theta\:=const$$

where $\:{\epsilon\:}_{0}$ denotes the preset incidence bias of the lifting wing, $\:{\upepsilon\:}$ denotes the wing incidence angle relative to the airframe and K represents the incidence control gain.

**Fig. 12:**
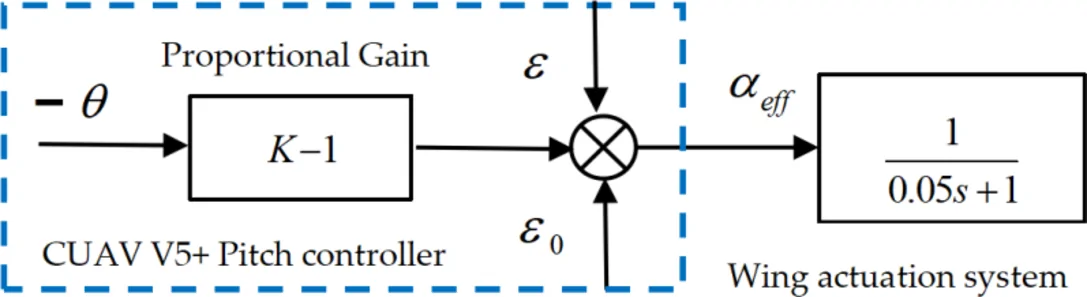
Schematic of the AoA control principle for the variable-incidence wing.

Figure 12 illustrates the schematic of this decoupled control principle. The CUAV V5 + controller captures the real-time pitch angle $\:\theta\:$. The inverse of this angle $\:-\theta\:$ is multiplied by a proportional gain $\:\mathrm{K}-1\:$and summed with the preset bias $\:{\epsilon\:}_{0}$ to generate the final incidence command. Subsequently, this command drives the wing actuation system, which is mathematically modeled as a first-order inertial link $\:\left(1/(0.05\mathrm{s}+1)\right)$ to ensure smooth servo responses and filter out high-frequency mechanical jitter. Consequently, this feedforward control architecture effectively isolates the wing aerodynamic characteristics from the fuselage attitude.

Flight tests utilized optimized control parameters of $\:{{\upepsilon\:}}_{0}$ = 0 and *K* = 1.5. Under this configuration, when the UAV cruises at 10 m/s with an optimal pitch angle of −20°, the mechanism automatically maintains the target 10° wing AoA, precisely replicating the optimal results determined by CFD. Conversely, at other flight velocities (e.g., 8 m/s and 12 m/s), the wing AoA cannot reach the optimal value of 10°, thereby affecting the aerodynamic performance.

#### Field experiments and flight conditions

**Fig. 13:**
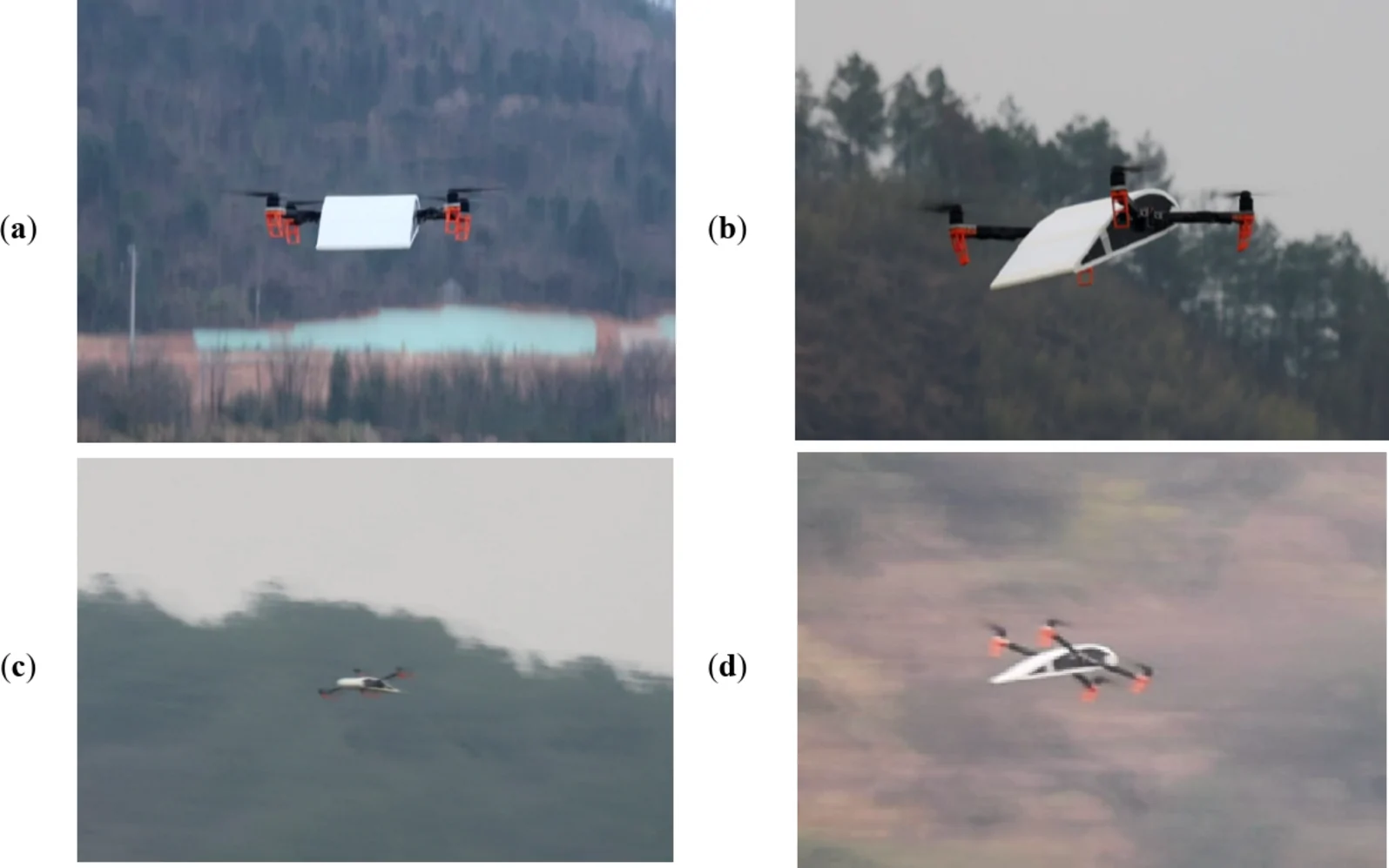
Field flight tests of the prototype variable-incidence lifting-wing quadrotor drone: (**a**) Takeoff and landing phase; (**b**) Stable hovering; (**c**) Horizontal forward flight (View 1); (**d**) Horizontal forward flight (View 2).

Field verification tests were conducted in a suburban area of Quzhou City, Zhejiang Province, China (Coordinates: 29.0053° N, 118.7976° E). The flight tests took place under cloudy conditions with an ambient temperature of 27 °C. To minimize environmental disturbances, testing was restricted to periods of light wind, with measured ground-level wind speeds ranging between 1 and 2 m/s. The flight test campaign encompassed three primary phases: takeoff and landing (Fig. 13a), static hovering (Fig. 13b), and horizontal forward cruise (Fig. 13c and d). For the cruise configuration, forward flight tests were conducted at a consistent altitude of approximately 20–30 m. This altitude range was selected to ensure operation Out of Ground Effect (OGE) while facilitating effective visual observation. The UAV was evaluated at target cruising velocities of 8 m/s, 10 m/s, and 12 m/s. Under these specific operating conditions, the flight data recorded equilibrium fuselage pitch angle and flight velocity pairs of (− 15°, 8 m/s), (− 20°, 10 m/s), and (− 25°, 12 m/s), respectively. To ensure measurement reliability and positioning accuracy throughout the maneuvers, horizontal velocity, flight altitude, voltage, current, and pitch attitude was acquired using a CUAV V5 + flight controller integrated with a NEO 3 GPS module and a Power Management Unit (PMU) power module.

#### Flight results

Multiple independent trials characterized the flight verification process, a strategy adopted to ensure the statistical reliability and repeatability of the results. The flight profile for each trial commenced with a stable hover at about 20 m to establish a reference baseline, followed by a transition to forward flight where the UAV accelerated to target cruising velocities of 8, 10, and 12 m/s. To maintain high data fidelity, specific exclusion criteria were implemented to filter outliers:

(1) **Coupled Instability**: Occurrences of significant coupling oscillations between pitch attitude and flight altitude;

(2) **Velocity Tracking Error**: Deviation of the maintained velocity from the setpoint by ± 0.2 m/s;

(3) **Insufficient Duration**: A steady-state flight phase duration of less than 8 s;

(4) **Signal Divergence**: Substantial deviations in telemetry (voltage, current, pitch, altitude, predicted AoA) from the steady-state average.

Upon filtering outliers, three valid datasets were retained for the final performance analysis.

**Fig. 14:**
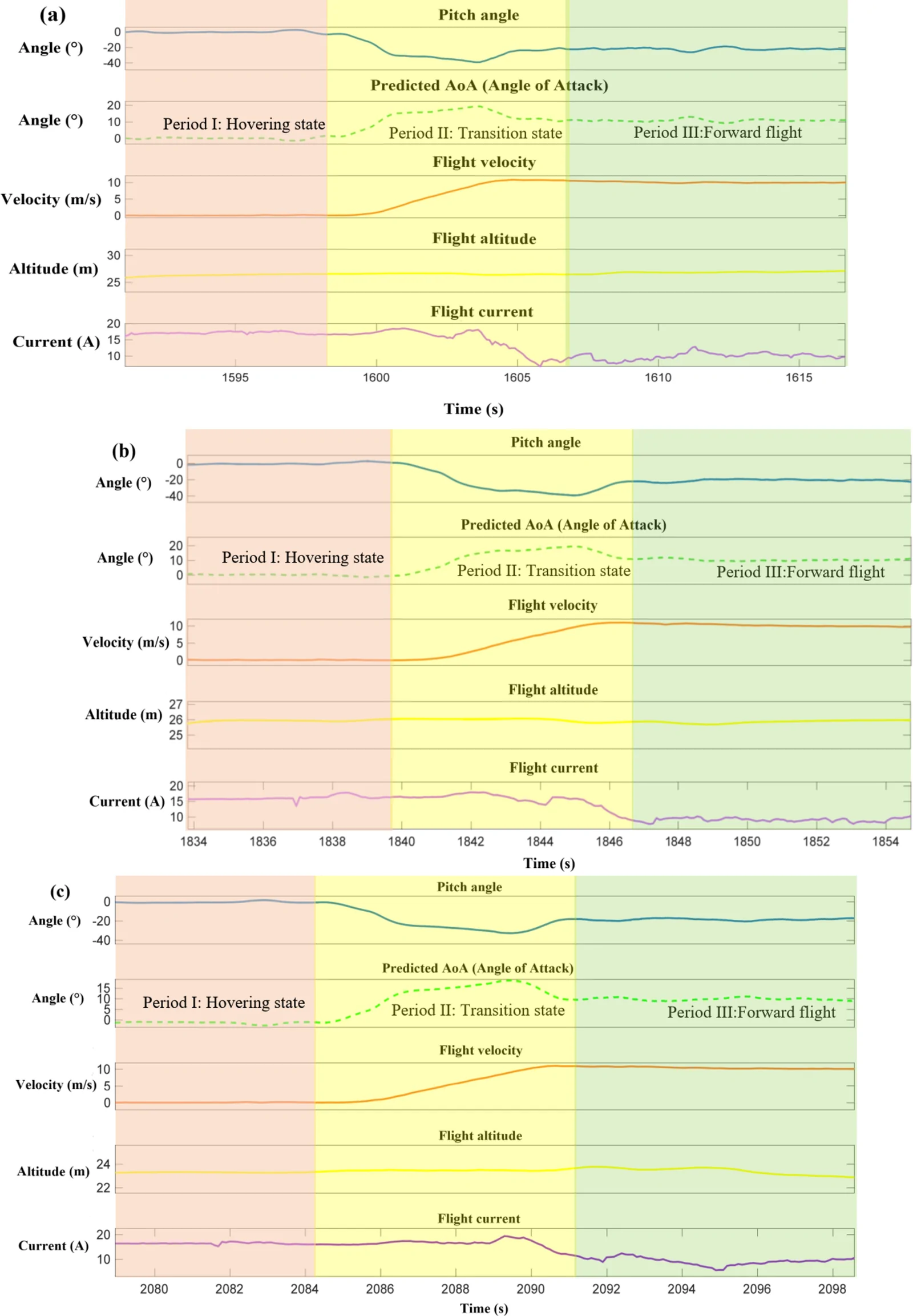
In-flight demonstrating the power reduction capabilities of the prototype variable-incidence UAV. Subplots (**a**), (**b**), and (**c**) present data from three independent flight test trials, supporting the repeatability of the results. Each panel records the temporal evolution of fuselage pitch angle, predicted wing Angle of Attack (AoA), flight velocity, altitude, and total flight current. The background colors denote three distinct operational phases: hovering (Period I), transition (Period II), and steady forward flight at 10 m/s (Period III), clearly highlighting the significant reduction in current consumption once wing-borne lift is established.

Experimental validation of the optimal flight conditions identified in the CFD analysis of Sect. 2.4 (velocity: 10 m/s; pitch: −20°; AoA: 10°) is presented in Fig. 14. This figure plots the time histories of pitch attitude, predicted AoA, forward velocity, flight altitude, and flight current during the transition from hover to steady-state cruise. It is noted that, due to strict payload weight constraints, direct telemetry instrumentation for propeller rotational speed (RPM) was not integrated; consequently, the evaluation of aerodynamic efficiency relies on the total electrical power consumption derived from the voltage and current measurements summarized in Table 3. Analysis of this telemetry data reveals critical characteristics regarding flight stability and aerodynamic efficiency. Specifically, during the transition phase from hover to forward cruise, the flight controller successfully maintained a constant altitude of approximately 20–30 m. This performance supports the system’s robust stability despite the dynamic coupling effects induced by rapid variations in pitch angle and velocity. Furthermore, a distinct correlation between fuselage attitude and energy consumption was observed: as the pitch angle *θ* optimized from the hovering state of 0° to the cruise configuration of − 20°, the current consumption decreased significantly from a baseline of 17.28 A to a cruise level of 9.79 A. This substantial reduction in power demand verifies the effective lift-generating capability of the wing at the optimal pitch angle.

The flight power consumption metrics summarized in Table 3 provide quantitative validation of the aerodynamic performance predicted by the numerical simulations. A distinct monotonic decrease in power demand is observed as the UAV transitions from hovering to the forward cruise regime. Specifically, the average power consumption drops from a baseline of approximately 399 W in the hover state to 227 W at a velocity of 10 m/s. This substantial attenuation corroborates the CFD analysis in Sect. 2.4, which identified 10 m/s as the optimal aerodynamic operating point where the lifting wing generates maximum efficiency. Furthermore, the experimental data at 12 m/s reveals a continued reduction in power to approximately 203 W. This trend demonstrates the unique advantage of the variable-incidence mechanism: unlike fixed-wing quadrotors that suffer from lift degradation due to negative pitch angles at high speeds, the proposed mechanism actively decouples the fuselage attitude from the wing Angle of Attack. This allows the wing to maintain high lift generation across a broader velocity envelope (8–12 m/s), significantly offloading the propulsion system and verifying the effectiveness of the proposed configuration in extending flight endurance. Furthermore, considering the aforementioned geometric offset caused by the uncompensated downwash angle ($\:{\alpha\:}_{ind\:}$), the reported 42.98% power saving is likely a conservative baseline. Implementing a closed-loop downwash compensation algorithm in future iterations could potentially yield even higher aerodynamic efficiencies by significantly aligning $\:{\alpha\:}_{eff\:}$with the optimal 10°.

**Table 3:** Measured flight power consumption of the prototype UAV across varying forward flight velocities.

Trial No.	Flight velocity (m/s)	Average voltage (V)	Average current (A)	Average flight power (W)
1, 2, 3	Hovering	23.12 ± 0.28	17.28 ± 0.53	400.48 ± 10.00
4, 5, 6	8 m/s	22.95 ± 0.17	13.29 ± 0.25	305.07 ± 5.13
7, 8, 9	10 m/s	23.26 ± 0.08	9.79 ± 0.40	227.77 ± 9.61
10, 11, 12	12 m/s	22.42 ± 0.11	10.89 ± 0.14	244.15 ± 2.04

Note: Total power includes flight controller, motors, and servos. Data are presented as Mean ±Standard Deviation (SD) based on three valid independent trials for each flight condition.

#### Flight endurance prediction and analysis

Based on the battery capacity of the proposed variable-angle lifting-wing UAV shown in Table 1, the flight power consumption at 10 m/s provided in Table 3, and the battery depth of discharge (DoD), the flight endurance and range predictions of the UAV are expressed as Eqs. (5) and (6).

6 $$\:{Time}_{\mathrm{p}\mathrm{r}\mathrm{e}}=\frac{{E}_{\mathrm{t}\mathrm{o}\mathrm{t}\mathrm{a}\mathrm{l}}\cdot \:DoD}{{P}_{cruise}}\times\:60$$

where $\:{E}_{\mathrm{t}\mathrm{o}\mathrm{t}\mathrm{a}\mathrm{l}}$ is the battery capacity (222 Wh), $\:\mathrm{D}\mathrm{o}\mathrm{D}$ presents the battery depth of discharge and $\:{P}_{cruise}$ represents the average flight power consumption at 10 m/s.

7 $$\:{Range}_{\mathrm{p}\mathrm{r}\mathrm{e}}=\frac{{Time}_{\mathrm{p}\mathrm{r}\mathrm{e}}}{60}\times\:10\times\:3.6$$

Here, $\:{Time}_{\mathrm{p}\mathrm{r}\mathrm{e}}$ denotes flight endurance of the proposed drone and $\:{Time}_{\mathrm{p}\mathrm{r}\mathrm{e}}$ is the range prediction of the proposed drone.

**Fig. 15:**
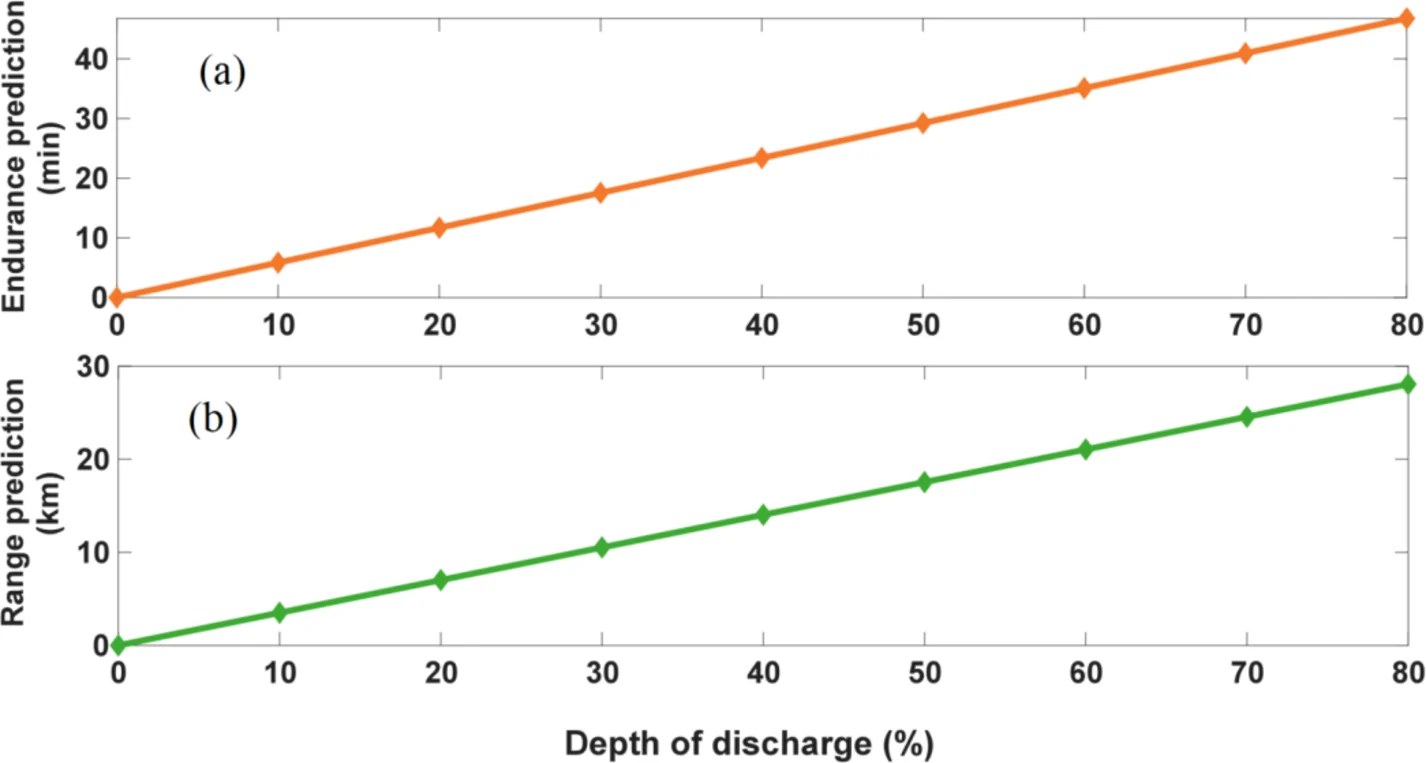
Predictive analytical results of (**a**) flight endurance and (**b**) maximum range for the proposed UAV as a function of the battery’s Depth of Discharge (DoD), calculated at a constant cruising speed of 10 m/s.

As illustrated in Fig. 15, predictive analyses of the proposed UAV’s flight endurance and range as a function of the allowable battery depths of discharge (DoD) were conducted based on Eqs. (6) and (7). According to Table 1, the system utilizes a 6S2P 21,700-type lithium-ion battery pack with a total capacity of 222 Wh. Furthermore, Table 3 indicates that the average flight power consumption of the UAV at a cruising speed of 10 m/s is 227.77 W. Focusing on practical operational limits designed to balance performance and battery health, the allowable DoD is typically restricted to 80%. Under this condition, the maximum flight endurance of the UAV is 46.78 min, and the corresponding maximum flight range is 28.07 km.

The aerodynamic efficiency advantage of the proposed variable-incidence lifting wing is quantified using the Power Saving Ratio $\:{\eta\:}_{gain}$. Based on the analytical flight power consumption established in Eq. (8), this metric reflects the relative power reduction achieved during forward cruise compared to the hovering baseline $\:{\eta\:}_{hover}$. Accordingly, the efficiency gain is defined by the following expression:

8 $$\:{\eta\:}_{gain}=\frac{{P}_{hover}-{P}_{cruise}\left(V\right)}{{P}_{hover}}\times\:100\mathrm{\%}$$

Here, $\:{\eta\:}_{gain}$ represents the normalized efficiency gain, $\:{\mathrm{P}}_{\mathrm{h}\mathrm{o}\mathrm{v}\mathrm{e}\mathrm{r}}$ denotes the average power consumption measured during the static hovering state, $\:{P}_{cruse}\left(V\right)$ corresponds to the average power consumption required at a specific forward flight velocity $\:\mathrm{V}$.

The Power Saving Ratio $\:{\eta\:}_{gain}$ serves as a normalized metric to quantify the aerodynamic efficiency enhancements achieved by the variable-incidence lifting-wing configuration during forward flight, relative to a static hovering baseline. Mathematically defined as the percentage difference between the mean power consumption in hover $\:{P}_{hover}$ and that at a specific cruising velocity $\:{P}_{cruse}\left(V\right)$, this formulation effectively isolates the contribution of lift generation to energy conservation, independent of the absolute power magnitude. From an engineering perspective, the metric offers both high intuitiveness and a direct correlation with flight endurance capabilities. Furthermore, the inherent normalization facilitates the direct quantification of efficiency gain, thereby enabling valid cross-platform comparisons of aerodynamic performance among different UAVs.

**Fig. 16:**
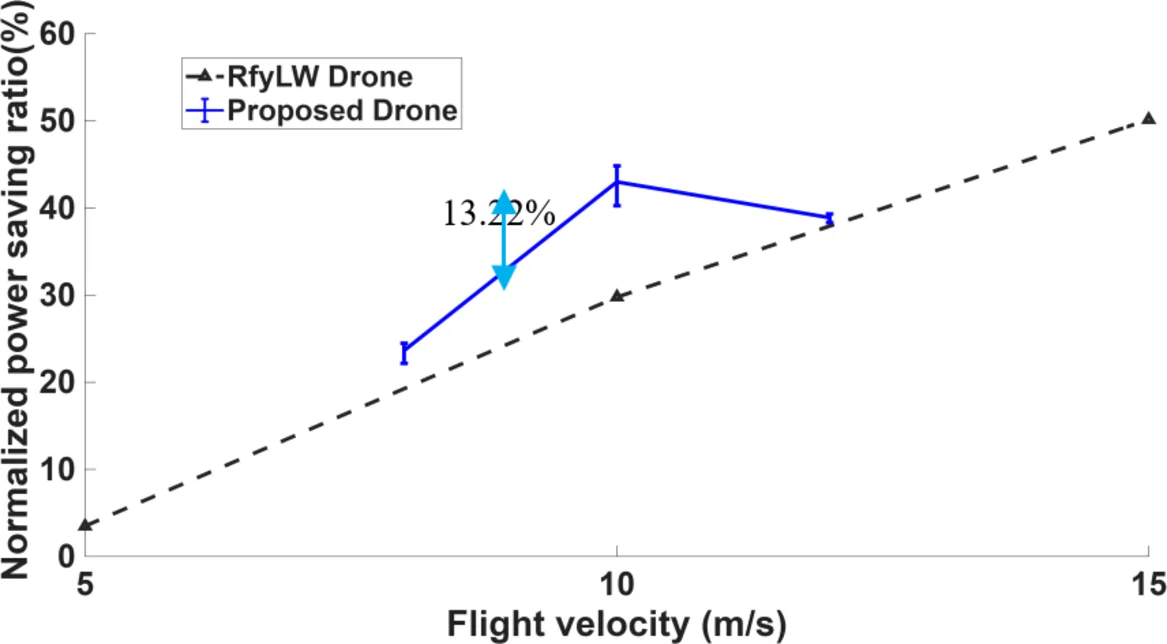
Comparison of the normalized power saving ratio between the proposed variable-incidence UAV and the RflyLW^8,21,25^ drone across varying flight velocities.

Figure 16 illustrates the comparative evaluation of the normalized power saving ratio between the proposed variable-incidence prototype and the RflyLW benchmark across the flight velocity spectrum. It is evident that the proposed UAV consistently exhibits superior energy efficiency within the tested range of 8 to 12 m/s. Notably, at the critical cruising velocity of 10 m/s, the proposed drone achieves a power saving ratio of 43.22%, representing a significant net improvement of 13.22% over the RflyLW drone. This performance advantage directly validates the aerodynamic characteristics analyzed in Sect. 2.4. Specifically, the variable-incidence mechanism effectively decouples the fuselage attitude from the wing AoA. By maintaining the wing at its optimal high-lift AoA rather than suffering from the lift-degrading negative pitch attitudes inherent to fixed-wing quadrotors like the RflyLW, the proposed system maximizes aerodynamic lift generation and substantially reduces the propulsion load.

## Conclusions

This study addresses the inherent aerodynamic inefficiencies of conventional lifting-wing quadrotors during variable-speed forward flight by proposing a novel variable-incidence lifting-wing mechanism. By actively decoupling the aerodynamic wing Angle of Attack (AoA) from the fuselage pitch attitude via dynamic incidence adjustments, the proposed design ensures optimal lift generation and minimizes aerodynamic drag across a broad flight envelope. Comprehensive evaluations via CFD simulations and prototype flight tests yield the following key conclusions:

- **Aerodynamic Decoupling and Optimization**: Numerical analyses reveal that the optimal aerodynamic performance is achieved at a fuselage pitch angle of − 20° combined with a wing AoA of 10°–12°. Flow visualization supports that the variable-incidence mechanism effectively suppresses boundary layer separation on the wing’s upper surface and mitigates detrimental rotor wake interference, which typically occurs at excessive pitch angles (exceeding − 25°).
- **Significant Energy Efficiency Enhancement**: Flight validation demonstrates substantial energy savings during forward cruise. Compared to the static hovering baseline power consumption of 400.48 W, the prototype operating at cruising velocities of 8 m/s, 10 m/s, and 12 m/s recorded significantly reduced power demands of 305.07 W, 227.77 W, and 244.15 W, respectively. Notably, at the optimal cruising speed of 10 m/s, the drone achieves a maximum normalized power saving ratio of 42.98%.
- **Superiority Over Fixed-wing Configurations**: A comparative analysis validates the superiority of the variable-incidence strategy. At a 10 m/s cruising speed, the proposed prototype delivers an additional 13.22% improvement in power reduction efficiency compared to the original fixed-wing RflyLW drone, effectively overcoming the lift degradation associated with negative pitch attitudes in conventional designs.
- **Extended Flight Endurance and Range**: Leveraging the significantly reduced power consumption at the optimal 10 m/s cruise state, analytical predictions demonstrate that the UAV can achieve a prolonged flight endurance of 46.78 min and a maximum flight range of 28.07 km (under a conservative 80% battery Depth of Discharge limit). This highlights the substantial practical value of the variable-incidence design.
- **Limitations and Future Work**: While this study validates the aerodynamic and energy-saving benefits of the variable-incidence quadrotor, certain limitations remain. The current flight tests were conducted under mild environmental conditions using a simplified feedforward control strategy that approximates the induced downwash effect. Future research will focus on developing closed-loop adaptive control algorithms integrating real-time airspeed and downwash sensing. Furthermore, the UAV’s performance will be evaluated under more complex meteorological conditions, such as turbulent wind gusts.

Ultimately, this work provides a highly efficient and structurally viable solution for extending the flight endurance of high-performance VTOL UAVs, highlighting its promising application in long-duration missions deployed in confined, semi-enclosed, or energy-constrained environments, including infrastructure inspection, environmental monitoring, and post-disaster search and rescue^39^.

## Computational methods

### Computational model and domain

**Fig. 17:**
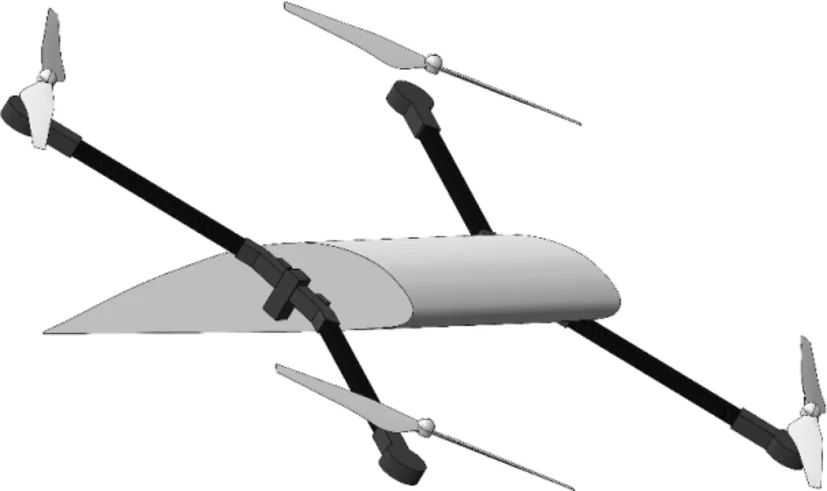
Aerodynamic model of the proposed variable-incidence lifting-wing quadrotor drone.

The simplified aerodynamic model of the proposed variable-incidence lifting-wing quadrotor is illustrated in Fig. 17. The model geometry comprises four rotors, rotor arms, and a centrally mounted lifting wing.

**Fig. 18:**
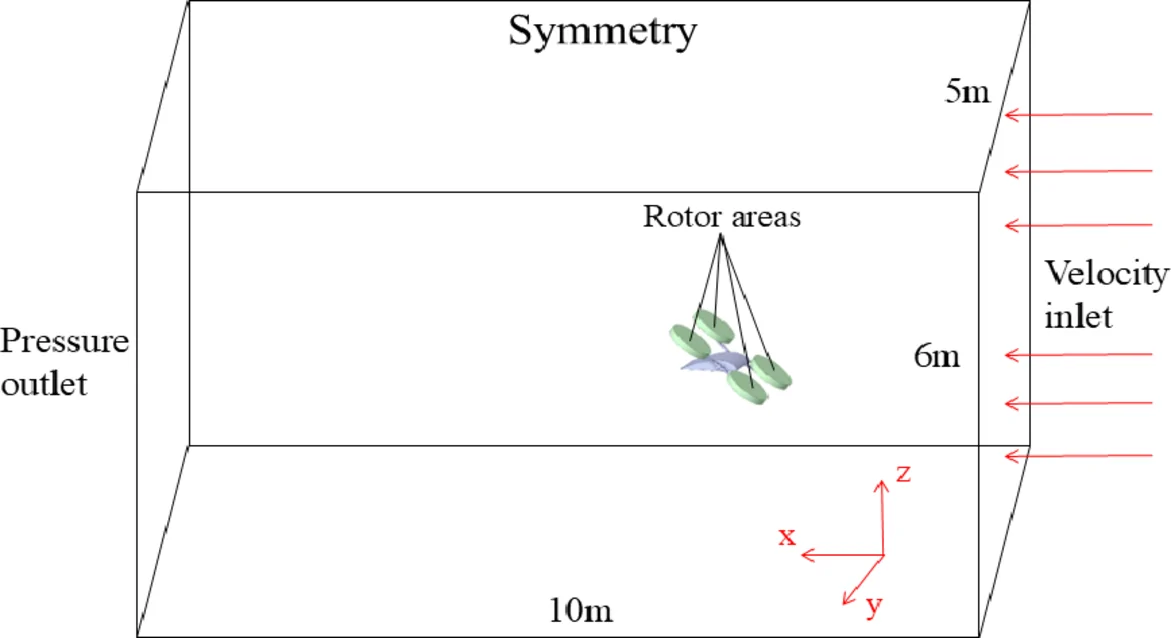
Full-flow computational domain applied for the drone system.

Due to the specific kinematic configuration of the quadrotor—where diagonal propellers counter-rotate—the flow field does not exhibit rotational symmetry. Consequently, simplified periodic boundary conditions are inapplicable. Therefore, a full-domain simulation approach was adopted. As shown in Fig. 18, the computational domain is partitioned into a stationary external fluid domain and distinct rotational domains enclosing each rotor.

The external computational domain is defined as a rectangular volume with dimensions of 10 m ($\:\mathrm{x}$) × 5 m ($\:\mathrm{y}$) × 6 m ($\:\mathrm{z}$). These dimensions were selected based on established guidelines^31,37,38^, ensuring the domain boundaries extend approximately 10 times the characteristic length of the UAV in the streamwise ($\:\mathrm{x}$) and vertical ($\:\mathrm{z}$) directions, and 5 times the wingspan in the spanwise ($\:\mathrm{y}$) direction. This sizing effectively minimizes boundary effects on the internal flow field solution.

### Boundary conditions

Given that the maximum flight velocity $\:{V}_{\mathrm{a}}$ is less than 20 m/s with Mach number ≤ 0.3, the airflow within the computational domain is modeled as an incompressible fluid. Consequently, the boundary conditions were configured as follows:

- **Inlet**: A velocity-inlet boundary condition was assigned to the upstream boundary surface.
- **Outlet**: The downstream far-field boundary was designated as a pressure-outlet with a gauge pressure of 0 Pa, simulating ambient atmospheric conditions.
- **Solid Surfaces**: A no-slip wall boundary condition was enforced on all solid surfaces, including the fuselage, wing, and rotors, to account for fluid viscosity and boundary layer attachment.
- **Far-field Boundaries**: All remaining outer boundaries of the fluid domain were set to symmetry boundary conditions. This setup effectively simulates a zero-shear, free-slip environment, representing flight in an unconfined open airspace.

### Reynolds number estimation

The Reynolds number (*Re*), represents the ratio of inertial forces to viscous forces within a fluid, defined by the following equation:

8 $$\:\mathrm{R}\mathrm{e}=\frac{{{\uprho\:}\mathrm{V}}_{\mathrm{a}}\mathrm{d}}{{\upmu\:}}=\frac{{\mathrm{V}}_{\mathrm{a}}\mathrm{d}}{\upsilon\:}$$

Where $\:\rho\:$ is the air density (kg/m^3^), $\:{\mathrm{V}}_{\mathrm{a}}$ is the airflow velocity (m/s), * d* denotes the wing chord length (mm), $\:\mu\:$ is the dynamic viscosity ($\:{P}_{a}\cdot\:s$), and $\:\nu\:$ is the kinematic viscosity (m^2^/s).

In this study, the wing chord length is set at 510 mm due to the structural constraints of the UAV. Under low-speed conditions, the air density $\:\rho\:$ and kinematic viscosity $\:\nu\:$ are treated as constants. Based on Eq. (8), the Reynolds number for the UAV in this research is determined to be 520,000.

### SST k-ω model

The governing equations were solved using a pressure-based coupled solver, which is particularly robust for establishing pressure-velocity coupling in incompressible and low-speed compressible flows characteristic of UAV flight. To account for turbulence effects, the Shear Stress Transport SST k-ω turbulence model was adopted. This model was selected for its superior capability in accurately predicting flow separation and resolving boundary-layer behavior under adverse pressure gradients-phenomena that critically influence the aerodynamic performance of lifting surfaces^17,31,38,39^.

Regarding spatial discretization, the second-order upwind scheme was applied to the convection terms of the momentum, turbulent kinetic energy, and specific dissipation rate equations to ensure high numerical accuracy. To guarantee the reliability of the computational results, a stringent convergence criterion was enforced, requiring the scaled residuals for all governing equations to fall below 10^− 6^.

### Model mesh design

**Fig. 19:**
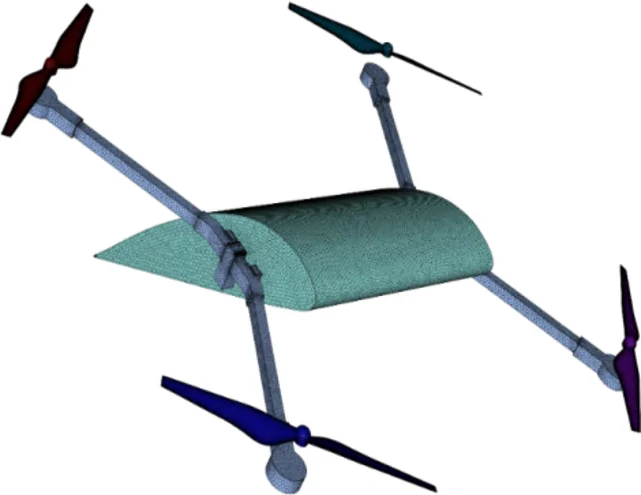
Surface meshing applied to the aerodynamic model of the proposed variable-incidence lifting-wing quadrotor drone.

The computational grid for the aerodynamic model was generated using ANSYS Fluent Meshing 2024 R1. As illustrated in Fig. 19, a hybrid meshing strategy was employed to discretize the domain. Unstructured polyhedral elements were utilized for the complex geometries of the fuselage, wing, and rotational domains to ensure geometric fidelity and high mesh quality, while preserving computational efficiency.

**Fig. 20:**
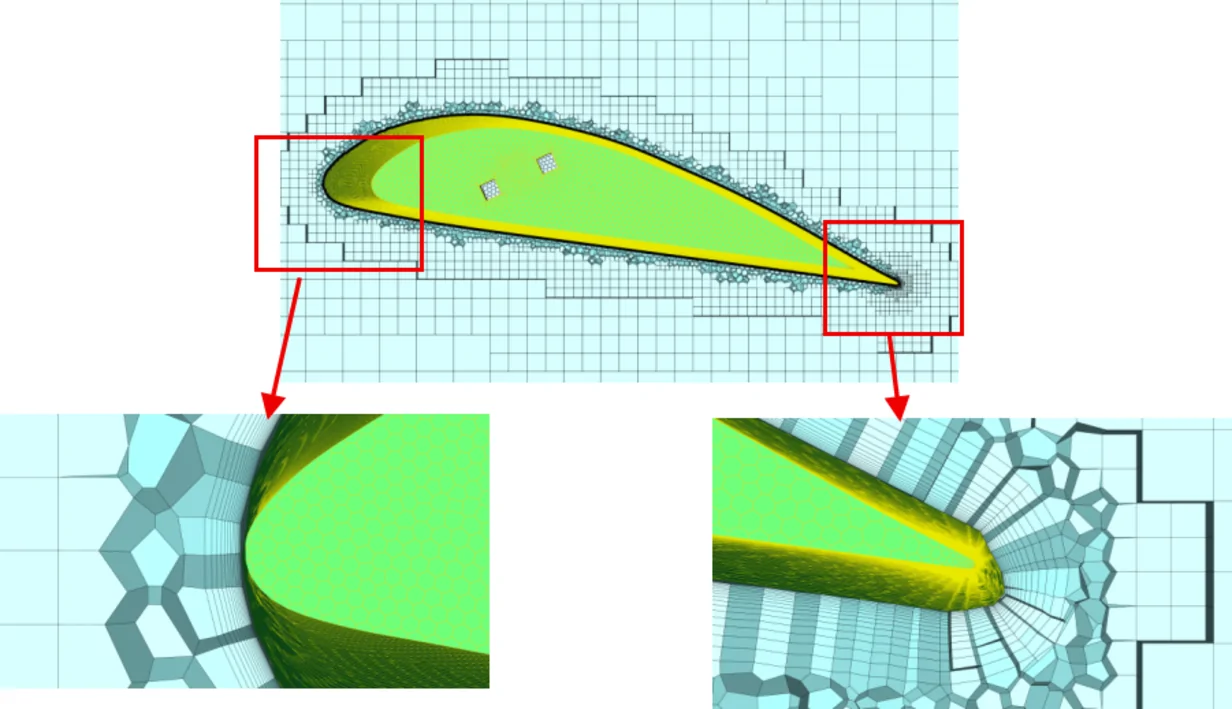
Applied airfoil boundary layer meshing.

Accurate prediction of aerodynamic forces, particularly the lift and drag acting on the lifting wing, necessitates high-resolution discretization of the near-wall flow field. Consequently, a dedicated boundary layer mesh was constructed on the wing surface, as shown in Fig. 20. To comply with the requirements of the SST k-ω turbulence model for low-Reynolds-number wall treatment, the mesh was refined to achieve a non-dimensional wall distance (y+) of approximately 1. Specifically, the height of the first prismatic layer was set to 0.05 mm. A total of 15 inflation layers were generated with a smooth growth rate to effectively capture the viscous boundary layer profile and ensure a seamless transition from the near-wall prism layers to the core polyhedral mesh.

### Verification of mesh independence and mesh quality analysis

To eliminate numerical discretization errors, a mesh independence study was conducted. The lift force of the lifting wing was computed under a fixed operating condition, $\:\alpha\:\:=\:{10}^{^\circ\:}$, $\:\theta\:\:=\:-{25}^{^\circ\:}$, and $\:{\mathrm{V}}_{\mathrm{a}\mathrm{i}\mathrm{r}}=12\:\mathrm{m}/\mathrm{s}$, using grid sizes ranging from 5 million to 12 million elements.

**Fig. 21:**
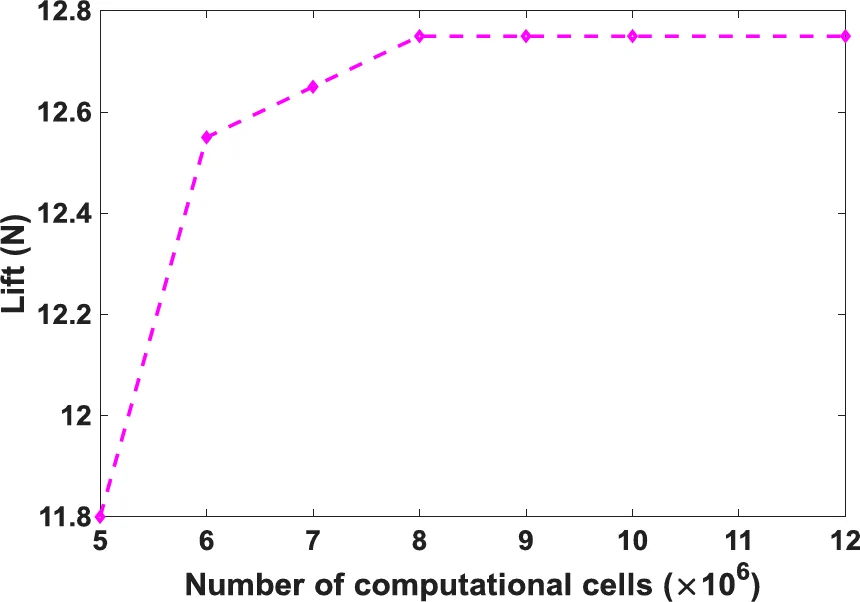
Lift computed for the lifting wing as a function of the number of computational cells applied in the flow field model.

Figure 21 illustrates the variation of computed lift force with respect to the grid resolution. The results indicate that the solution converges when the grid size reaches approximately 8 million elements, beyond which further refinement yields negligible changes in the calculated lift. Consequently, the grid configuration with 8 million elements was selected for all subsequent simulations to balance computational accuracy and cost.

**Fig. 22:**
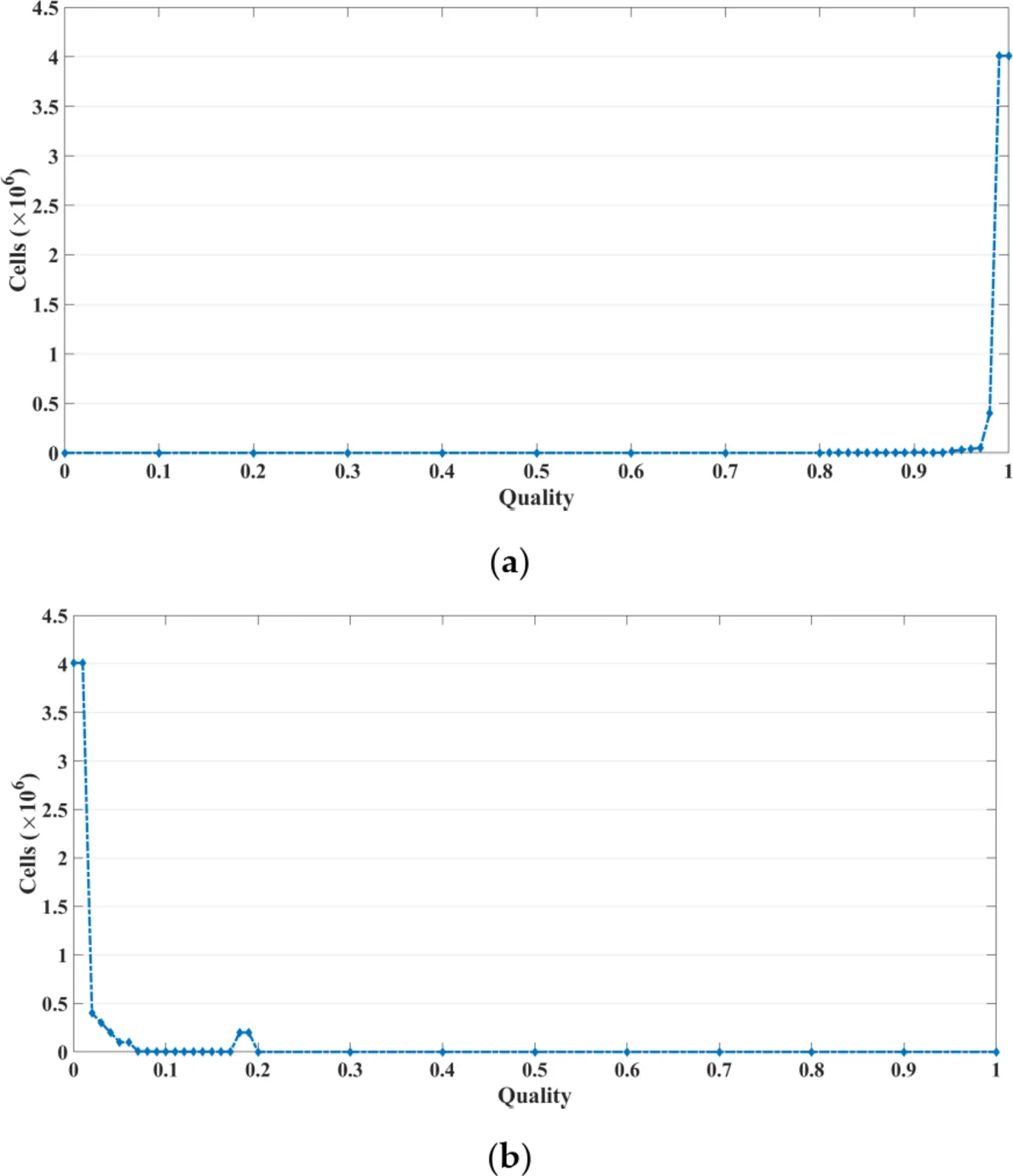
Mesh quality analyses relative to the number of computational cells: (**a**) orthogonal quality; (**b**) skewness.

Following the selection of the optimal grid size, the geometric quality of the final volume mesh was rigorously evaluated using orthogonal quality and skewness metrics, as shown in Fig. 22a and b, respectively.

**Orthogonal Quality**: The mesh exhibited an average orthogonal quality of 0.967, with values ranging from 0.085 to 1.0. Notably, only six cells fell into the lower quality range.

**Skewness**: The average skewness was 0.0135, with a maximum value of 0.84.

These metrics demonstrate that the vast majority of mesh elements possess highly regular geometric configurations. Such high-quality discretization is conducive to numerical stability and convergence, particularly when employing the SST k-ω turbulence model.

### Validation of computational methodology

A validation experiment was conducted to assess the fidelity of the numerical framework employed in this study, wherein the CFD-predicted thrust of four rotor assemblies was compared with experimental measurements.

The combined numerical simulation and experimental validation approach is widely recognized as the gold standard for aerodynamic performance evaluation of novel VTOL aircraft^29,40,41^. Accordingly, a validation experiment was conducted to assess the fidelity of the numerical framework employed in this study, wherein the CFD-predicted thrust of four rotor assemblies was compared with experimental measurements.

**Fig. 23:**
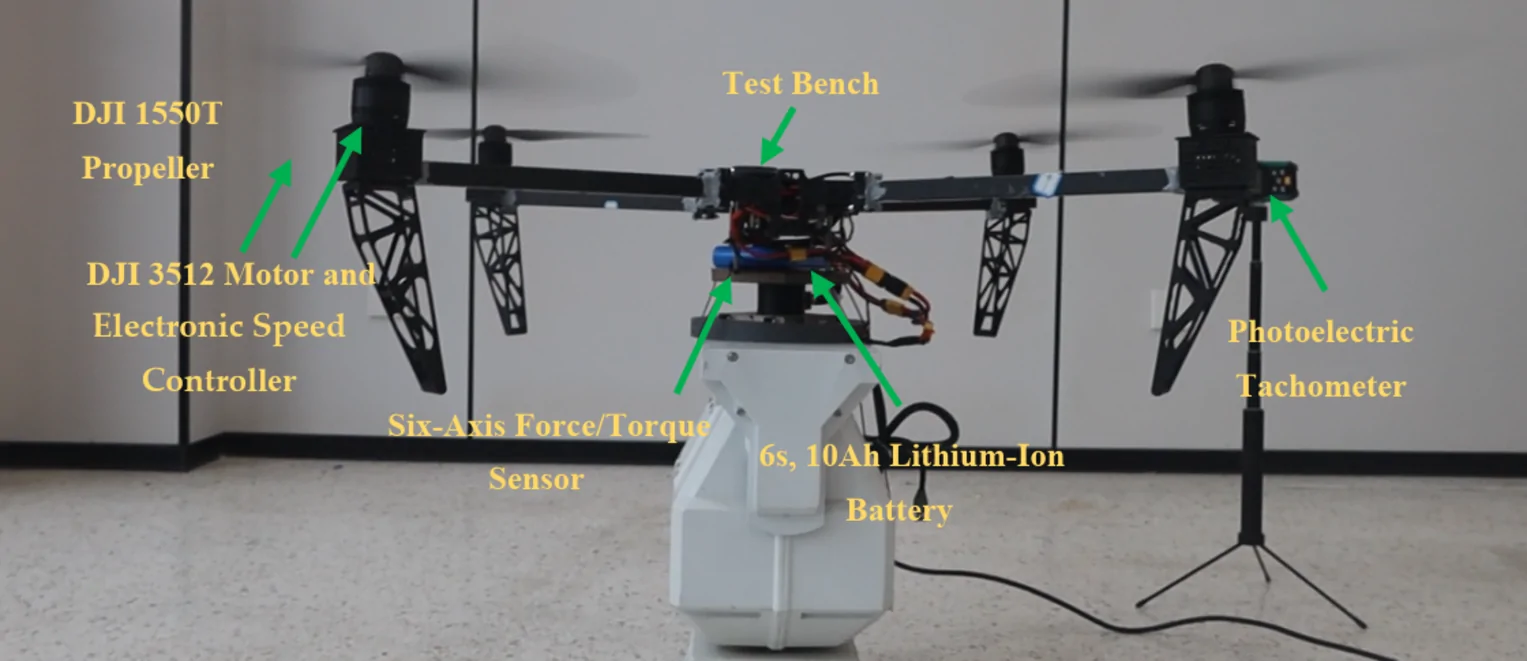
Experimental propeller test bench employed for validation tests.

Although this validation primarily targets the rotor aerodynamics, the strong agreement between the predicted and experimental values establishes confidence in the chosen turbulence model, mesh strategy, and solver settings. Experimental data were acquired using the propeller test bench illustrated in Fig. 23. The experimental setup comprised four DJI 3512 KV460 brushless direct current (BLDC) motors driven by a high-capacity 6 S lithium-ion battery (25.2 V, 10 Ah, 50 A maximum discharge current). This power source ensured a stable voltage output, thereby minimizing experimental errors induced by voltage fluctuations. Key instrumentation included a photoelectric tachometer for rotational speed measurement and an FC-Medusa-U six-axis force sensor for thrust data acquisition. The specific propellers tested were the DJI 1550 T, identical to those used in the simulation model.

While the static hover validation establishes confidence in the baseline rotor aerodynamics, mesh strategy, and solver settings, it inherently lacks the capacity to directly validate the complex rotor-wake and wing interference present in forward flight. Conducting high-fidelity wind tunnel tests equipped with a six-axis balance for the full vehicle in forward flight represents a substantial experimental challenge and falls outside the scope of this initial study. Therefore, to corroborate the fidelity of the numerical framework under forward flight conditions, this study relies on established precedents in the literature. Previous studies investigating rotor-on-wing interference and compound VTOL aerodynamics^17,29,31,38^ have consistently demonstrated that the SST k − ω turbulence model, combined with high-resolution polyhedral meshing, accurately captures flow separation, wake trajectories, and aerodynamic force variations in similar dynamic environments. Building upon these validated methodologies, our CFD approach is considered sufficiently reliable for identifying the optimal aerodynamic operating points.

**Fig. 24:**
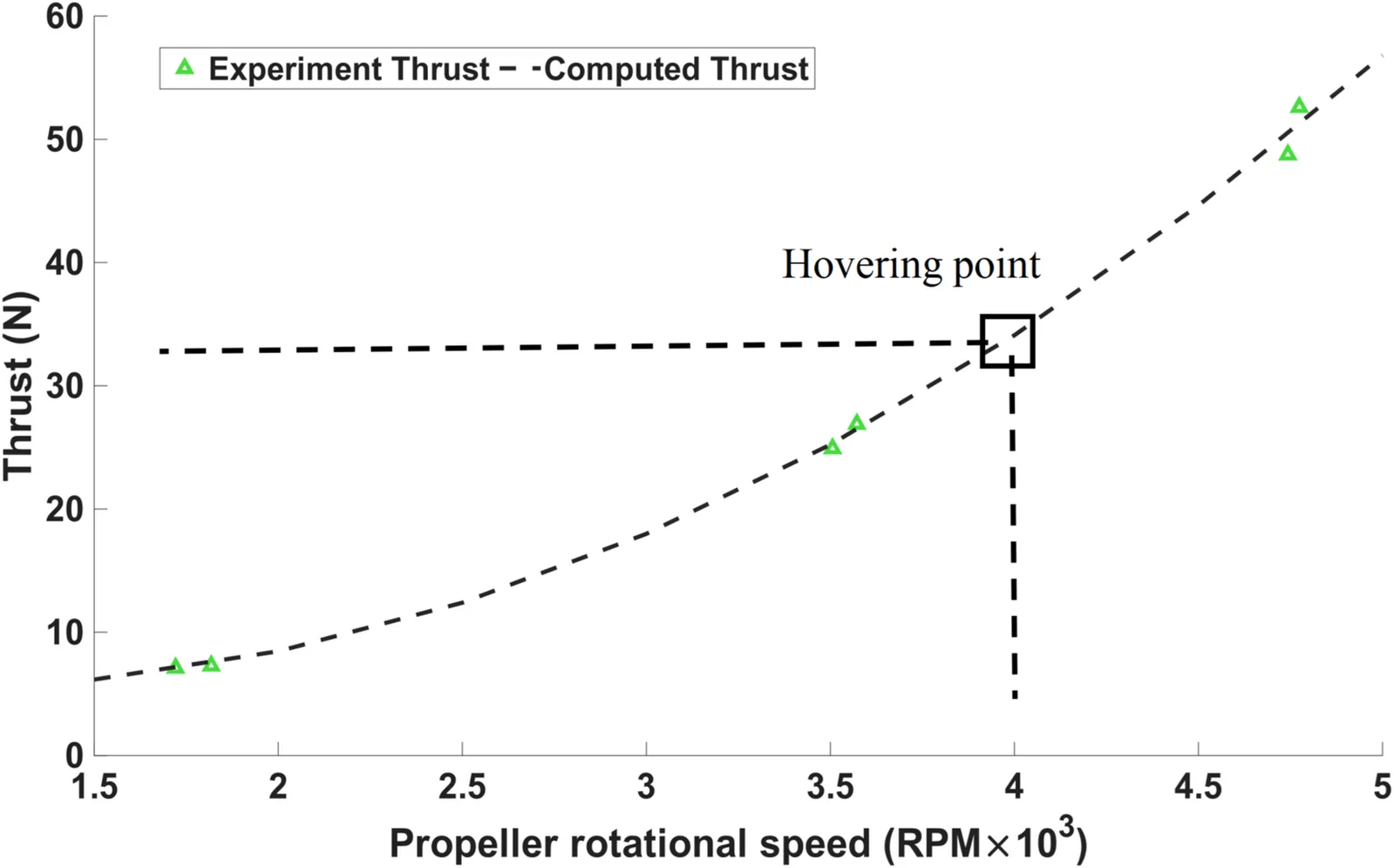
Comparison of computed and experimental thrust values plotted versus propeller rotational speed.

Figure 24 compares the computed and experimental thrusts across various rotational speeds, including a numerical quadratic fit. The excellent agreement validates the numerical framework. For the proposed 3.4 kg UAV, the equilibrium hovering point requires a total thrust of 33.32 N, achieved experimentally at approximately 4001 rpm. This validated condition establishes a critical boundary for subsequent full-vehicle hovering simulations.

## Supplementary Information

Below is the link to the electronic supplementary material.

Supplementary Material 1

## Data Availability

The datasets generated and/or analysed during the current study are available from the corresponding author on reasonable request.
